# Synthesis of double perovskite and quadruple perovskite nanocrystals through post-synthetic transformation reactions†

**DOI:** 10.1039/d2sc00574c

**Published:** 2022-03-30

**Authors:** Hanjun Yang, Tong Cai, Lacie Dube, Ou Chen

**Affiliations:** Department of Chemistry, Brown University 324 Brook St. Providence Rhode Island 02912 USA ouchen@brown.edu

## Abstract

Lead-free halide perovskite nanocrystals (NCs) represent a group of emerging materials which hold promise for various optical and optoelectronic applications. Exploring facile synthetic methods for such materials has been of great interest to not only fundamental research but also technological implementations. Herein, we report a fundamentally new method to access lead-free Bi-based double perovskite (DP) and quadruple perovskite (or layered double perovskite, LDP) NCs based on a post-synthetic transformation reaction of Cs_3_BiX_6_ (X = Cl, Br) zero-dimensional (0D) perovskite NCs under mild conditions. The produced NCs show good particle uniformity, high crystallinity, and comparable optical properties to the directly synthesized NCs. The relatively slow kinetics and stop-on-demand feature of the transformation reaction allow real-time composition–structure–property investigations of the reaction, thus elucidating a cation-alloyed intermediate-assisted transformation mechanism. Our study presented here demonstrates for the first time that post-synthetic transformation of 0D perovskite NCs can serve as a new route towards the synthesis of high-quality lead-free perovskite NCs, and provides valuable insights into the crystal structures, excitonic properties and their relationships of perovskite NCs.

## Abstract 2

Lead-free perovskite nanocrystals are synthesized by post-synthetic transformation reactions. The post-synthetic transformations show the structural flexibility of zero-dimensional perovskite nanocrystal materials.

## Graphical abstract

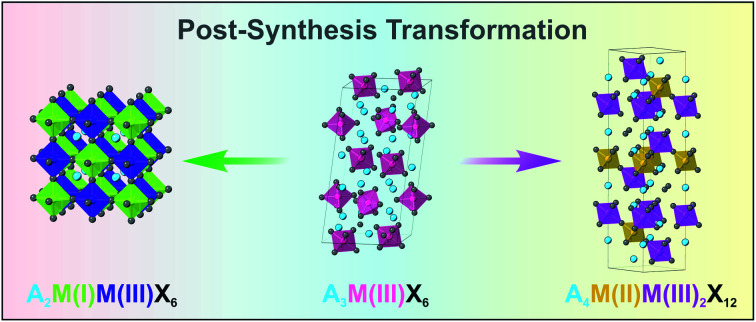

## Introduction

**Scheme 1:**
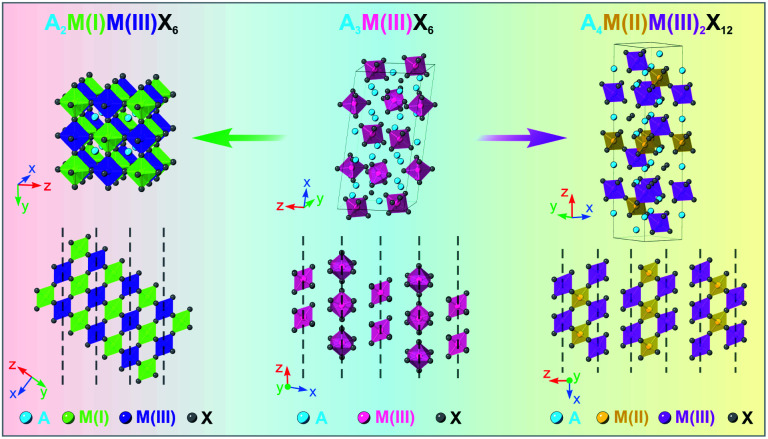
Schematic illustration of the crystal structures of the A_2_M(i)M(iii)X_6_ DP phase (left), A_3_M(iii)X_6_ 0D perovskite phase (middle), and A_4_M(ii)M(iii)_2_X_12_ LDP phase (right).

Moving away from lead-based perovskite materials driven by environmental and toxicity concerns, lead-free halide perovskite (LFHP) nanocrystals (NCs) have emerged as a promising family of materials that exhibits great potential in various applications owing to their unique optoelectronic properties and highly adaptable solution processibility.^1–8^ Within the family, double perovskites (DPs) and layered double perovskites (LDPs, also known as quadruple perovskites) are two widely-studied types of LFHPs with high structural similarity to lead-based perovskites in the cubic phase (Scheme 1).^9–14^ By replacing every two Pb^2+^ cations in APbX_3_ lead-halide perovskites with a pair of M(i) and M(iii) cations, the DP crystal structure with balanced charge can be formed with a general chemical formula of A_2_M(i)M(iii)X_6_ (A = Rb^+^, Cs^+^, *etc.*; X = Cl^−^, Br^−^ or I^−^). The resulting DP lattice is composed of alternating [M(i)X_6_]^5−^ (M(i) = Na^+^, K^+^, Ag^+^, *etc.*) and [M(iii)X_6_]^3−^ (M(iii) = In^3+^, Sb^3+^, Bi^3+^, *etc.*) octahedra which construct a cubic framework through corner-shared halide X^−^ anions (Scheme 1, left).^10^ One step further, when replacing every two M(i) cations in the DP with one M(ii) cation and one vacancy (V), the LDP crystal structure with a chemical stoichiometry of A_4_M(ii)M(iii)_2_X_12_ can be obtained with an ordered sandwich structure of M(iii)–M(ii)–M(iii) layers between two adjacent vacancy layers along the [111] direction of the original DP lattice (Scheme 1, right).^13^ Intriguing optical and optoelectronic properties have been reported for DP and LDP NCs.^15–19^ For example, Cs_2_M(i)InCl_6_ DP NCs, such as Cs_2_KInCl_6_ NCs and Cs_2_(Na_1−*x*_Ag_*x*_)InCl_6_ NCs, show efficient white light photoluminescence (PL) through the recombination of the self-trapped excitons (STEs) due to the strong exciton–phonon coupling (EPC) strength.^20–23^ Vacancy-ordered double perovskites Cs_2_M(iv)X_6_, including Cs_2_SnX_6_ and Cs_2_ZrX_6_ NCs, exhibit a unique thermally activated delayed fluorescence with a tunable PL profile and high PL quantum yields (>60%).^24–26^ The Cs_4_CuSb_2_Cl_12_ LDP NCs possess a characteristic narrow direct band gap of ∼1.8 eV, making them a promising candidate material for applications such as photovoltaics, photoconductors and photocatalysts.^27–29^

With regard to the material fabrication, current synthetic approaches that produce high-quality DP or LDP NCs mostly rely on a solution-based hot-injection method, where the needed precursors are rapidly mixed in a preheated organic solution to initiate the NC nucleation and growth.^30–32^ Yet, unlike lead-halide perovskite NCs, injection-based syntheses of DP and LDP NCs have encountered more problems due to the involvement of increased types of components as well as other possible reaction side-products, oftentimes leading to poor purities in the product composition and/or crystal phase.^33–35^ To alleviate this problem, indirect synthesis strategies for halide perovskite NCs involving converting the pre-existing NCs to targeted materials with desired compositions and crystal phases have been proposed and demonstrated.^36–41^ In this regard, zero-dimensional (0D) perovskite NCs with isolated metal-halide [MX_6_]^3−^ octahedra have been prototyped as an ideal starting material for transformation reactions (Scheme 1, middle).^42,43^ The metal-deficient stoichiometry of 0D perovskites offers a strong tendency of transforming to other perovskite-type phases upon external stimulations such as varying solvent polarity or introducing additional metal halide precursors.^44–56^ For example, the transformation reaction of Cs_4_PbX_6_ 0D perovskite NCs to obtain uniform CsPbX_3_ 3D perovskite NCs has been successfully demonstrated.^44,57–59^ Unlike injection-based syntheses that rely on the rapid nucleation and growth rate to obtain NCs, the slow reaction kinetics and stop-on-demand feature of post-synthesis transformation are more beneficial for obtaining intermediate species during structural and compositional evolution. In addition, without using high temperature and reactive reagents, the post-synthesis transformation reaction could also be more suitable to obtain unstable species. Recently, we reported a colloidal synthesis of high-quality Cs_3_BiX_6_ (X = Cl, Br) 0D perovskite NCs which can be further transformed into Cs_3_Bi_2_X_9_ layered perovskite NCs post synthesis.^50^ To date, however, synthesizing lead-free DP or LDP NCs through a facile 0D-perovskite NC transformation reaction has not yet been reported.

Herein, we present a fundamentally new approach to access two important classes of LFHP NCs, *i.e.*, Cs_2_AgBiX_6_ (X = Cl, Br) DP NCs and Cs_4_MBi_2_Cl_12_ (M = Cd, Mn) LDP NCs through post-synthetic transformation of Cs_3_BiX_6_ 0D perovskite NCs by taking advantage of their structural flexibility and compositional tunability. The resulting LFHP NCs exhibit comparable qualities in terms of particle morphological uniformity and optical performances to the ones synthesized directly. Importantly, unlike the fast reactions during direct syntheses, the more controllable and slow reaction nature of the post-synthetic transformation process allows us to closely monitor the reaction evolution and capture intermediate products. Thus, the reaction mechanism can be studied and delineated as an alloy-intermediate-assisted intra-particle transformation process for both DP and LDP cases. In all, our study provides not only a new route towards fabrication of high-quality LFHP NCs, but also provides a powerful platform which enables a deeper understanding of composition–structure–property relationships of such fascinating materials.

## Results and discussion

Zero-dimensional (0D) Cs_3_BiX_6_ (X = Cl, Br) NCs were first synthesized using our previously reported method.^50^ The crystal structure is composed of isolated [BiX_6_]^3−^ octahedra (Scheme 1, middle), leading to a 0D electronic structure with molecule-like optical properties.^60,61^ The resulting 0D NCs showed characteristic sharp absorption peaks at 333 nm and 381 nm for Cs_3_BiCl_6_ and Cs_3_BiBr_6_ perovskite NCs, respectively (Fig. S1a†). Neither Cs_3_BiCl_6_ nor Cs_3_BiBr_6_ 0D perovskite NCs exhibited any detectable PL signals at room temperature. The X-ray diffraction (XRD) patterns of both samples confirmed the monoclinic crystal structure of 0D perovskite (Fig. S1b†).^50^ Both Cs_3_BiCl_6_ and Cs_3_BiBr_6_ 0D perovskite NCs exhibited a cubic shape with an average edge length of 7.7 ± 1.0 nm and 8.9 ± 1.3 nm, respectively (Fig. S1c–h†). The obtained high-quality Cs_3_BiX_6_ NCs were used as the starting material for the post-synthetic transformation reactions described below.

**Fig. 1:**
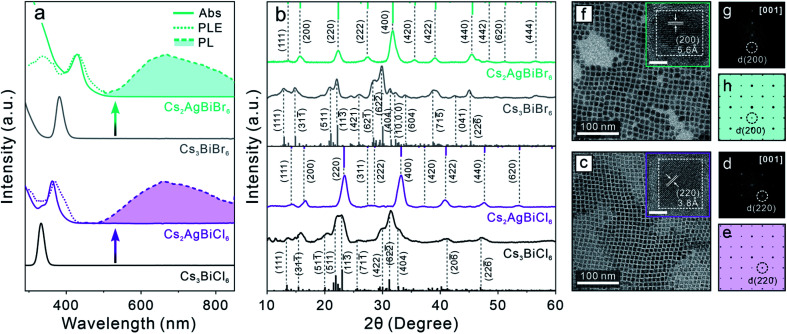
(a) Absorption spectra (solid lines), PL spectra (dashed lines with shade) and PLE spectra (dotted lines) of starting 0D perovskite NCs and the Cs_2_AgBiX_6_ DP NCs obtained by the transformation reactions. (b) XRD patterns of the starting 0D perovskite NCs and the Cs_2_AgBiX_6_ DP NCs. (c and f) TEM images of the resulting Cs_2_AgBiCl_6_ (c) and Cs_2_AgBiBr_6_ (f) DP NCs. Insets: HR-TEM images of the corresponding DP NCs. Scale bar = 5 nm. (d and g) Fast-Fourier transformation (FFT) patterns of the corresponding HR-TEM images. (e and h) Simulated electron-diffraction patterns of the DP crystal structure along the [001] zone axis.

Post-synthetic transformation reaction from Cs_3_BiX_6_ 0D perovskite NCs to Cs_2_AgBiX_6_ 3D DP NCs was carried out by adding an acetonitrile solution of silver nitrate (AgNO_3_) to the Cs_3_BiX_6_ NC hexane dispersion (see ESI† for details). The reaction was conducted at 50 °C for 24 hours and monitored by UV-vis absorption and PL spectroscopies. The low miscibility between hexane and acetonitrile facilitates the separation of NCs from the AgNO_3_ precursor, and the reaction temperature was chosen to avoid solvent evaporation. The occurrence of Cs_3_BiX_6_ NC transformation reaction was proved by the emergence of a new absorption peak at 362 nm (430 nm) and gradual disappearance of the initial 333 nm (381 nm) absorption peak for the Cl-based (Br-based) sample (Fig. 1a). In addition, a broad PL peak centered at 672 nm (670 nm) with a full-width-at-half-maximum (FWHM) of 0.69 eV (0.63 eV) was detected (Fig. 1a), which can be assigned to the STE emission of the Cs_2_AgBiCl_6_ (Cs_2_AgBiBr_6_) DP NCs.^16^ The PL excitation (PLE) spectra overlapped with the absorption profiles of the final Cs_2_AgBiX_6_ DP NC samples, which unambiguously confirmed that the PL peaks originate from the band gap absorption of Cs_2_AgBiX_6_ (Fig. 1a). The small shoulders of the PLE spectra on the longer wavelength side were likely caused by fine electronic substructures near the band edge of the Cs_2_AgBiX_6_ DP NCs.^62^ These optical properties of the Cs_2_AgBiX_6_ NCs synthesized *via* the post-synthesis transformation reaction showed nearly no difference from the ones synthesized *via* the direct hot-injection method (Fig. S2†).

XRD patterns of the final samples confirmed the structural transformation from the 0D monoclinic phase to the 3D cubic DP phase (Fig. 1b). All the Bragg diffraction peaks of the final product matched the standard XRD pattern of the cubic DP structure. The disappearance of the characteristic 0D perovskite diffraction features – for example the absence of signals in the 2*θ* range of 30.0–32.8° corresponding to the (422) (622̄) and (404) characteristic Bragg diffractions of the monoclinic 0D phase (Fig. 1b) – proved the completion of transformation reaction, in accordance with optical measurements (Fig. 1a). Crystallite sizes of the produced DP NCs were estimated by Scherrer analyses to be 7.9 ± 1.0 nm and 7.8 ± 1.3 nm for Cs_2_AgBiCl_6_ and Cs_2_AgBiBr_6_ NCs, respectively (Fig. S3, Tables S1 and S2†), in agreement with TEM measurements (Fig. 1c, f and S4†). High-resolution TEM (HR-TEM) images and the corresponding fast-Fourier transformation (FFT) patterns of both samples showed the DP lattice viewed along its [001] zone direction (Fig. 1d, e, g and h).^31,39^ Nearly unchanged particle size and morphology between the starting and the final NCs suggested the preservation of individual NC integrity during the transformation reaction.^44^

**Fig. 2:**
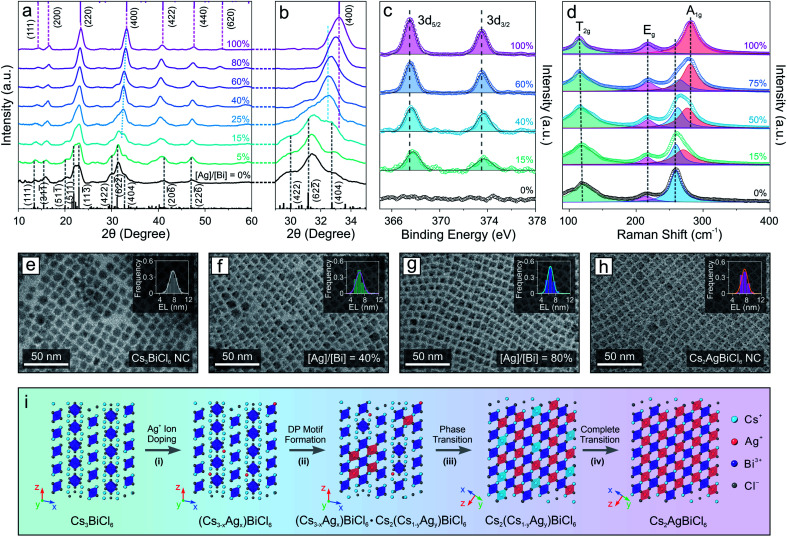
(a) XRD evolution of the DP transformation reaction from the Cs_3_BiCl_6_ 0D perovskite NCs. Black bars represent the Cs_3_BiCl_6_ standard peak positions, and the purple bars represent the Cs_2_AgBiCl_6_ standard peak positions. (b) Zoomed-in area of the XRD patterns for clear visualization. (c) XPS spectra of Ag for the NCs during the transformation reaction. (d) Raman spectra of the NCs during the transformation reaction. (e–h) TEM images of the starting Cs_3_BiCl_6_ 0D perovskite NCs, intermediate Cs_2_(Cs_1−*x*_Ag_*x*_)BiCl_6_ NCs, and the final Cs_2_AgBiCl_6_ NCs. Insets: size-distribution histograms (EL: edge length). (i) Schematic illustration of the proposed transformation reaction mechanism.

The drastic structural leap from 0D perovskite (with isolated octahedral units) to 3D perovskite (with connected octahedral units) motivated us to investigate the structural evolution mechanism. The relatively slow reaction kinetics and stop-on-demand feature of the transformation reactions allow us to fine-tune the Ag concentration as well as closely monitor the transformation process of the Cs_3_BiCl_6_ NC model system (Fig. 2), in contrast to the injection-based synthesis routes which exhibit rapid reactions kinetics that hinder real-time monitoring of the reaction progress. XRD measurements showed that with increasing the feeding amount of the Ag precursor (*i.e.*, AgNO_3_), the crystal structure gradually changed from the initial 0D monoclinic phase to the 3D cubic DP structure (Fig. 2a). The cubic DP crystal structure started to emerge when the stoichiometry ratio between silver and bismuth (*i.e.*, [Ag]/[Bi]) reached 15% (Fig. 2a, b and S5†). Given the low concentration of Ag^+^ cations, the Ag-doped 0D Cs_3_BiCl_6_ NCs coexisted with the Cs_2_(Cs_1−*x*_Ag_*x*_)BiCl_6_ alloyed DP NCs (with the M(i) sites occupied by either Cs^+^ or Ag^+^ cations). Upon further increasing the Ag precursor amount, the DP crystal diffraction features became prominent, revealing an increased contribution from the DP crystal phase (Fig. 2a and b). Besides, all the DP diffraction peaks shifted to larger 2*θ* angles, indicating a continuous lattice contraction during the reaction (Fig. 2b), in line with the insertion of small Ag^+^ cations (ionic radius: 1.15 Å) to the M(i) site and replacing the larger Cs^+^ cation (ionic radius: 1.67 Å).^63^ For instance, the (400) peak shifted from 32.6° to 33.2° (Fig. 2b, and S5, Tables S1 and S3–S7†), corresponding to an ∼1% lattice constant shrinkage from 10.9 Å to 10.8 Å. The insertion of Ag^+^ cations and the corresponding local structural change were further supported by the X-ray photoelectron spectroscopy (XPS) measurements, where the Ag 3d peaks (3d_5/2_ and 3d_3/2_) of the sample showed enhanced intensity with increasing the [Ag]/[Bi] ratio (Fig. 2c and S6†). An ∼0.3 eV downshift in binding energy suggested an increase of the Ag–Cl bond strength,^64^ corresponding to the evolution from the Ag-doped 0D Cs_3_BiCl_6_ phase (where Ag^+^ cations occupy the original Cs-sites with the resulting Ag–Cl bond length of ∼3.7 Å) to the Cs_2_(Cs_1−*x*_Ag_*x*_)BiCl_6_ alloyed DP phase (Ag–Cl bond length ∼ 2.7 Å). Such gradual insertion of Ag^+^ cations was also confirmed by the energy dispersive X-ray spectroscopy measurements (Fig. S7 and S8†). This structural change during the transformation reaction can also be reflected by the Raman spectral evolution (Fig. 2d). The Raman peak at 259 cm^−1^ corresponds to the symmetric stretching mode (A_1g_) of the [BiCl_6_]^3−^ octahedra (Fig. S9†).^65,66^ After introducing the Ag component, a shoulder-like Raman feature emerged on the higher binding energy side, which was due to the Bi–Cl bond length shortening of the [BiCl_6_]^3−^ octahedra upon lattice contraction. With increasing Ag concentration, the intensity of the newly emerged peak became more pronounced along with a gradual intensity decrease of the initial Raman peak (Fig. 2d). In addition, the new Raman peak continuously shifted to a larger wavenumber from 274 cm^−1^ to 281 cm^−1^, suggesting a Bi–Cl bond shortening process (Fig. 2d). Such results agreed well with the XRD measurements showing a continuous shrinkage of the [BiCl_6_]^3−^ octahedra upon increasing the Ag^+^ concentration (Bi–Cl bond length decreased from 2.72 Å to 2.68 Å, Fig. S5, Tables S1 and S3–S7†). Moreover, the peak of [BiCl_6_]^3−^ octahedral scissoring mode (T_2g_) became more defined (Fig. 2d), further supporting the increased crystallography symmetry with a simplified neighboring Cs^+^ environment (involved in the [BiCl_6_]^3−^ scissoring vibration mode) in the final DP structure.^66^ TEM images showed that the perovskite NCs kept a similar size and size distribution for all the Cs_2_(Ag_*x*_Cs_1−*x*_)BiCl_6_ NCs with different amounts of Ag, proving the preservation of NC integrity during the reaction (Fig. 2e–h and S10†). This observation indicated an intra-particle transformation mechanism in contrast to the dissolution–recrystallization process reported for the case of Cs_4_PbBr_6_ 0D perovskite NCs to CsPbBr_3_ perovskite NW transformation.^51^ Taking these results together, the transformation reaction can be described by the following chemical equations:

1 Cs_3_BiCl_6_ + *x*Ag^+^ → Cs_(3−*x*)_Ag_*x*_BiCl_6_ + *x*Cs^+^

2 2Cs_(3−*x*)_Ag_*x*_BiCl_6_ + (*y* − *x*)Ag^+^ → Cs_(3−*x*)_Ag_*x*_BiCl_6_·Cs_2_Cs_(1−*y*)_Ag_*y*_BiCl_6_ + (*y* − *x*)Cs^+^

3 Cs_(3−*x*)_Ag_*x*_BiCl_6_·Cs_2_Cs_(1−*y*)_Ag_*y*_BiCl_6_ + (*y* − *x*)Ag^+^ → 2Cs_2_Cs_(1−*y*)_Ag_*y*_BiCl_6_ + (*y* − *x*)Cs^+^

4 Cs_2_Cs_(1−*y*)_Ag_*y*_BiCl_6_ + (1 − *y*)Ag^+^ → Cs_2_AgBiCl_6_ + (1 − *y*)Cs^+^

The entire structural transformation process of Cs_3_BiCl_6_ 0D perovskite to Cs_2_AgBiCl_6_ 3D DP can be delineated as shown in Fig. 2i. Upon introducing the Ag^+^ cations, the Ag-doped 0D perovskite phase is formed initially with Ag^+^ occupying the Cs-sites (Fig. 2i, step (i)). Increasing the Ag concentration leads to the partial phase transition from the 0D A_3_BiCl_6_ phase to the alloyed DP phase where both [AgCl_6_]^5−^ and [CsCl_6_]^5−^ octahedral units are formed by sharing the corner Cl^−^ ions with the [BiCl_6_]^3−^ octahedra inside each NC (Fig. 2i, step (ii)). In this process, the cubic DP crystal motif emerges and coexists with the pristine 0D perovskite structure. With further increasing the Ag concentration, the intermediate Cs_2_(Ag_*x*_Cs_1−*x*_)BiCl_6_ alloyed DP crystal motif progressively dominates the crystal phase of the NCs (Fig. 2i, step (iii)). When the Ag concentration reaches a sufficient level to fully occupy the M(i) sites of the DP crystal structure, pure Cs_2_AgBiCl_6_ DP NCs are obtained as the final product (Fig. 2i, step (iv)). Similar crystal structural evolution was also observed when transforming Cs_3_BiBr_6_ perovskite NCs to Cs_2_AgBiBr_6_ DP NCs (Fig. S11†), further proving the proposed transformation mechanism. It is worth mentioning that recent theoretical studies showed that the monoclinic 0D perovskite structure of the Cs_3_BiCl_6_ is thermodynamically more stable than the Cs_2_CsBiCl_6_ cubic DP structure (where one third of Cs^+^ ions occupy the M(i) site of the cubic DP crystal lattice, Scheme 1).^67^ However, the experimental results demonstrated here reveal that by introducing a smaller monovalent cation, the thermodynamically unfavored DP structure with both cations (*e.g.*, Cs^+^ and Ag^+^) co-occupying the M(i) site of DP phase can be stabilized, which is at least partly because of the improved tolerance factor (*t*, increased from 0.78 for Cs_2_CsBiCl_6_ DP to 0.85 for Cs_2_AgBiCl_6_ DP).^68^

**Fig. 3:**
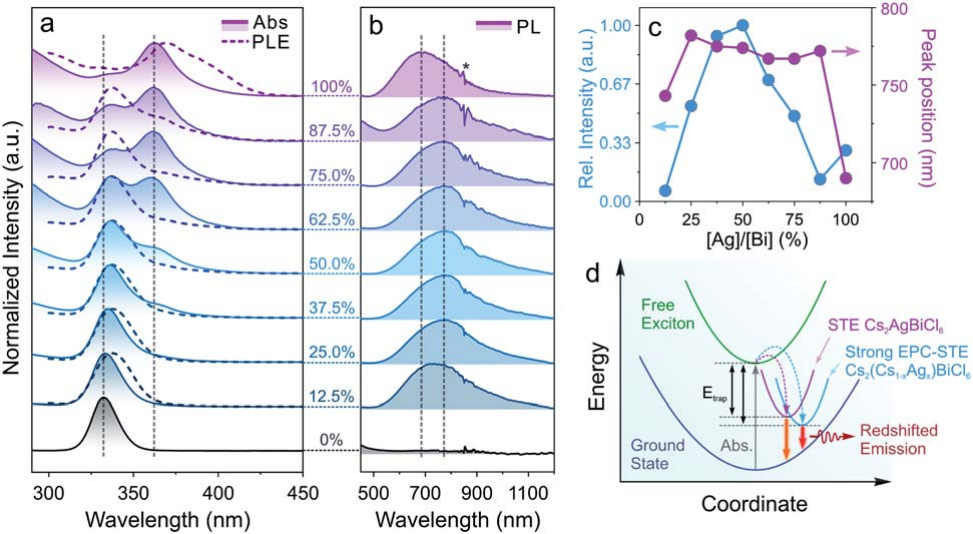
(a) Evolution of the absorption spectra (solid lines with shade) and the PLE (dashed lines) spectra of the Cs_2_(Cs_1−*x*_Ag_*x*_)BiCl_6_ NCs. (b) PL spectral evolution of the Cs_2_(Cs_1−*x*_Ag_*x*_)BiCl_6_ NCs (the asterisk marks the artifact caused by switching detectors). (c) The PL peak position and the relative PL intensity as a function of the [Ag]/[Bi] stoichiometry ratio. (d) Schematic illustration of the STE-based electronic transition and emission mechanism.

Doping of alkali metal cations (*e.g.*, Na^+^ or K^+^) into the M(i) site of DPs was utilized to tune and optimize the optical performances of DP NCs by lifting the parity forbidden selection rule and partially reducing the electronic dimensionality.^21,69^ However, such effect in the DPs doped with large monovalent cations such as Cs^+^ has been rarely reported due to synthetic challenges.^32,70,71^ The post-synthetic transformation reaction discussed above allows for the preparation of Cs_2_(Cs_1−*x*_Ag_*x*_)BiCl_6_ alloyed DP NCs, which leads us to further study the optical property evolution of the reaction. Upon increasing the Ag^+^ concentration, a new absorption peak at 362 nm (Fig. 3a) emerged along with a gradual decrease of the 333 nm peak from the initial Cs_3_BiCl_6_ NCs.^31,33^ Meanwhile, a broad emission feature centered at 765–785 nm (FWHM of 250–300 nm, or 0.55–0.65 eV) was observed for the Cs_2_(Cs_1−*x*_Ag_*x*_)BiCl_6_ intermediates (Fig. 3b), which can be assigned to the radiative recombination of the STEs evidenced by the large Stokes shift and wide emission profile.^72^ The energy transfer from [BiCl_6_]^3−^ is evident based on the PLE and absorption profile overlapping (Fig. 3a). The intensity of this emission peak increased drastically and reached its maximum at [Ag]/[Bi] = 50% (Fig. 3c). This initial PL intensity increase can be attributed to the increase of the Ag^+^ cation concentration, which can serve as centers for STE formation. Further increasing the Ag concentration ([Ag]/[Bi] > 50%) leads to a decrease of the PL intensity due to the enforced parity selection rule and the loss of dimension-reduction effect while forming DP lattices (Fig. 3c).^21,73^ In addition, the obvious ionic size difference between Cs^+^ (1.67 Å) and Ag^+^ (1.15 Å) can result in local structure deformation with an enhanced EPC effect, thus enlarging the STE trapping energy (*E*_trap_),^74^ which was proved by the increased Stokes shift of the intermediates as compared to that of the final Cs_2_AgBiCl_6_ DP NCs (2.1 eV *vs.* 1.6 eV, Fig. 3c and d). The enlarged *E*_trap_ favors the radiative recombination of STEs by suppressing the non-radiative detrapping processes, in good accordance with the PL intensity measurements as well as time-resolved PL lifetime study results (Fig. 3c, d, S12 and Table S8†).^75^ The longer emission lifetime (∼1 μs) of the Cs_2_(Ag_*x*_Cs_1−*x*_)BiCl_6_ intermediates than that of the final DP NCs supports the suppression of STE non-radiative detrapping-induced processes (Fig. S12 and Table S8†).^32^ Also, the Cs_2_(Cs_1−*x*_Ag_*x*_)BiCl_6_ alloyed DP NCs showed high stability as minimal variations in the absorption spectra were observed upon storage under ambient conditions for at least 42 days (Fig. S13†). No such red-shifted PL signal was observed for any intermediates (*i.e.*, Cs_2_(Cs_1−*x*_Ag_*x*_)BiBr_6_ alloyed DP NCs) in the Cs_3_BiBr_6_ transformation reactions (Fig. S14†), which can be attributed to the weaker EPC effect of the Br-based system as reported previously.^76,77^

**Fig. 4:**
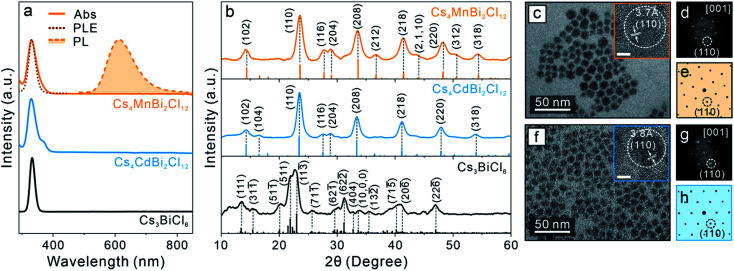
(a) Absorption (solid lines), PL (dashed line with shade) and PLE (dotted line) of the starting Cs_3_BiCl_6_ 0D perovskite NCs and the final LDP NCs obtained by the transformation reaction. (b) XRD patterns of the starting 0D perovskite NCs and the final LDP NCs. Bars represent standard diffraction peak positions. (c and f) TEM images of the final Cs_4_MnBi_2_Cl_12_ (c) and Cs_4_CdBi_2_Cl_12_ (f) LDP NCs. Insets: HR-TEM images of the corresponding LDP NCs. Scale bar = 5 nm. (d and g) The FFT patterns of the corresponding HR-TEM images. (e and h) Simulated electron diffraction patterns of the LDP crystal structure along the [001] zone axis.

To further generalize this post-synthetic transformation method to fabricate other types of LFHP NCs, we performed the transformation reaction targeting 2D LDP NCs. In this case, a biphasic reaction was performed by adding excess amounts of MnCl_2_ or CdCl_2_ metal halide powders to the Cs_3_BiCl_6_ NC colloidal solution (2 mg mL^−1^ in toluene) at 50 °C. The reaction was terminated after 24 hours, and the samples were purified and collected through centrifugation (see details in the ESI†). The UV-vis absorption peaks of the final Cs_4_MnBi_2_Cl_12_ and Cs_4_CdBi_2_Cl_12_ LDP NCs showed a subtle blueshift (∼3 nm) with direct band gaps of 3.38 eV (for Cs_4_MnBi_2_Cl_12_ LDP NCs) and 3.42 eV (for Cs_4_CdBi_2_Cl_12_ LDP NCs) determined by Tauc plot analyses (Fig. 4a and S15†), in good accordance with the samples directly synthesized using a hot-injection method.^30^ An appreciable peak broadening effect was observed for both cases (Fig. 4a), which may be attributed to the [BiCl_6_]^3−^ octahedral distortion induced by connecting to the neighboring [M(ii)Cl_6_]^4−^ (M(ii) = Mn or Cd) octahedral units through formation of the bridging Bi–Cl–M(ii) bonds (Fig. S16†).^30^ In addition, a weak PL peak at 614 nm (FWHM: 98 nm or 0.32 eV, PL quantum yield < 1%) was detected for the Cs_4_MnBi_2_Cl_12_ LDP NC sample (Fig. 4a). The corresponding PLE spectrum showed a good match with the sample absorption profile (Fig. 4a), proving that the energy transfer process from the [BiCl_6_]^3−^ (origin of the absorption) to the neighboring Mn^2+^ ion centers was responsible for the observed emission (^4^T_1g_ → ^6^A_1g_ electronic transition of the Mn^2+^ ion).^30,78,79^ Markedly, the absorption and PL spectra of the LDP NCs synthesized *via* the post-synthesis transformation method are comparable to those of the directly synthesized LDP NCs (Fig. S17†). XRD measurements of the final products confirmed the trigonal LDP crystal structure (space group: *R*3̄*m*) with the lattice parameters of *a* = 7.56 Å, *c* = 37.2 Å for Cs_4_MnBi_2_Cl_12_ and *a* = 7.60 Å, *c* = 37.2 Å for Cs_4_CdBi_2_Cl_12_ LDP NCs (Fig. 4b and S18, Tables S9 and S10†).^30,80^ TEM images showed that both Cs_4_MnBi_2_Cl_12_ and Cs_4_CdBi_2_Cl_12_ LDP NCs possessed a sphere-like shape with average diameters of 9.4 ± 1.6 nm and 10.8 ± 1.7 nm, respectively (Fig. 4c, f and S19†). The deviation from the cubic shape of starting Cs_3_BiCl_6_ NCs suggested an extensive reorganization of the crystal structure within individual NCs. HR-TEM images and the corresponding FFT patterns further confirmed the LDP structure with high crystallinity of the samples (Fig. 4d, e, g and h). Together, all these results unambiguously exhibited the successful syntheses of high-quality LDP NCs through the post-synthetic transformation from Cs_3_BiCl_6_ 0D perovskite NCs. Absorption spectra of the LDP NCs synthesized by transformation reaction showed no observable changes upon storage under ambient conditions for over a month, suggesting high stability of the NCs synthesized by this method (Fig S20†).

**Fig. 5:**
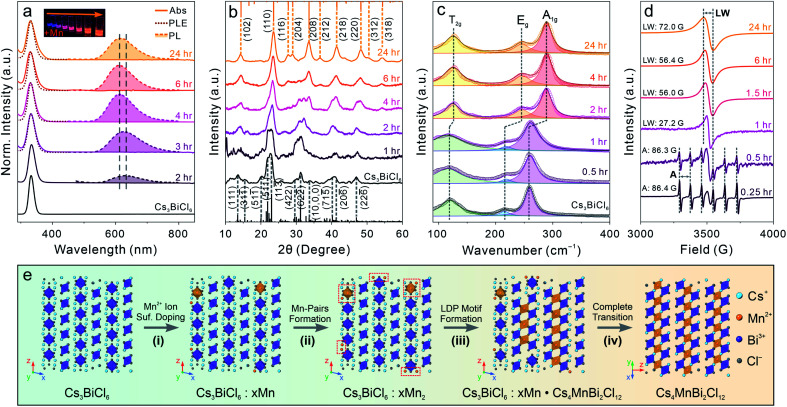
(a) Evolution of the absorption spectra (solid lines), PLE spectra (dotted lines), and PL spectra (dashed lines with shade). Inset: photograph of the NCs with different reaction times under UV light. (b) XRD pattern evolution during the transformation reaction. (c) Raman spectra of the NCs during the transformation reaction. (d) EPR spectral evolution during the transformation reaction. LW: linewidth; and *A*: hyperfine splitting constant. (e) Schematic illustration of the proposed transformation reaction mechanism.

To understand the detailed transformation mechanism, we monitored the optical property evolution for the Mn-containing reaction. The absorption peak showed both red-shifting and broadening effects as described for the final Cs_4_MnBi_2_Cl_12_ LDP NCs within 3 hours of the transformation reaction and remained nearly unchanged afterwards (Fig. 5a). Meanwhile, the PL peak started to emerge at around 2 hours followed by blue-shifting from ∼630 nm to ∼610 nm along with a dramatic intensity increase within 4 hours of the reaction (Fig. 5a and S21†). Both PL peak position and intensity showed negligible changes for the rest of the reaction (Fig. S21†). The PL peak blueshift indicated that the incorporated Mn ions migrated from the NC surface to the inside (diluting Mn^2+^ ions with reduced Mn–Mn coupling interactions) through a dopant inward diffusion process, similar to the post-synthetic Mn doping into CsPbCl_3_ NCs reported previously.^81^ Time-resolved PL lifetime measurements further supported the occurrence of inward diffusion by showing the average lifetime change from 0.25 ms for the 2 hour sample (relaxed spin-forbidden ^4^T_1g_ to ^6^A_1g_ transition by strong Mn–Mn coupling) to 0.44 ms (reenforced spin-forbidden transition after diluting the Mn^2+^ ion concentration), and finally to 0.25 ms for the final LDP NCs (intralayer antiferromagnetic ordering effect of the 2D LDP lattices) (Fig. S22 and Table S11†).^80,82^ The significantly longer PL lifetime (average PL lifetime of 0.25–0.44 ms for the post-synthesis transformed NCs *vs.* ∼ 10 μs for the directly synthesized ones) suggests that the defect-induced non-radiative recombination routes are greatly suppressed in the Cs_4_MnBi_2_Cl_12_ LDP NC synthesized *via* the post-synthesis transformation reaction as compared to the directly synthesized ones.^30^

XRD measurements showed that at the early stage of the reaction, the initial 0D perovskite remained as the dominant crystal phase, followed by a gradual transition to the Cs_4_MnBi_2_Cl_12_ LDP phase starting from 2 hours after the reaction started (Fig. 5b). This structural evolution was consistent with the optical measurements and was also supported by the Raman spectroscopy characterization (Fig. 5c). Raman spectra showed the emergence of a new Raman peak at a higher bonding energy of ∼290 cm^−1^ at around 2 hours of the reaction, which can be assigned to the symmetric stretching (A_1g_) of the Bi–Cl bonds with a shortened bond length of 2.62 Å in an octahedral coordination environment (Fig. S16†). The narrowing of Raman peaks, especially for the low-energy peak associated with the T_2g_ mode ([BiCl_6_]^3−^ scissoring vibration mode), reflected the high symmetry of the LDP crystal structure as compared to the Cs_3_BiCl_6_ structure.^66^ The electron paramagnetic resonance (EPR) spectra showed a six-fold hyperfine splitting pattern with an average splitting constant of ∼86.3–86.4 G at the initial reaction stage (within one hour), confirming that the Mn^2+^ ions were present in the isolated [MnCl_6_]^4−^ octahedral environment (replacing Bi^3+^ centers, Fig. 5d).^83,84^ The sextet pattern was later replaced by a single EPR peak with the linewidth gradually broadening from 27.2 G (at one hour) to 72.0 G (at 24 hours) for the final Cs_4_MnBi_2_Cl_12_ LDP NCs (Fig. 5d). We recently demonstrated that the symmetry-orientated spin-exchange interaction played the determining role in narrowing the EPR linewidth of Cs_4_(Cd_1−*x*_Mn_*x*_)Bi_2_Cl_12_ alloyed LDP NCs with increasing the Mn concentration.^30,85^ However, the opposite trend observed here indicated that the EPR linewidth evolution, in this case, was predominated by the Mn–Mn dipole–dipole interactions over the spin-exchange interactions.^30,85,86^ For the initial intermediate NCs, a strong localized spin-exchange interaction overwhelmed the global dipole–dipole interaction among the dispersed Mn^2+^ ion pairs, resulting in a relatively narrow EPR peak (Fig. 5d). Upon increasing the Mn concentration and transforming the crystal phase to the LDP structure containing in-plane [MnCl_6_]^4−^ octahedral layers (aligned in the (001) plane of LDP), the Mn–Mn dipole–dipole interactions enhanced and gradually outperformed the spin-exchange interactions, subsequently leading to a broadened EPR linewidth as observed in the experiments (Fig. 5d).

Taking all the results together, a complete transformation scheme to LDP NCs can be proposed as shown in Fig. 5e. Initially, the Mn^2+^ ions from the dissolved MnCl_2_ precursor (assisted by oleylamine ligands) reach surfaces of the starting Cs_3_BiCl_6_ NCs and subsequently form the inhomogeneously surface-doped Cs_3_BiCl_6_ NCs (replacing the surface Cs^+^ or Bi^3+^ ions, Fig. 5e, step (i)). When the Mn concentration is low, the Mn^2+^ ions preferentially replace the neighboring Cs^+^ ions (due to the relatively weak Cs–Cl bond) and occupy the A-site of the 0D perovskite NCs to form Mn–Mn pairs within proximity (shortest Mn–Mn distance of 4.6 Å *vs.* 7.5 Å in Cs_4_MnBi_2_Cl_12_ LDP) (Fig. 5e, step (ii)). The reaction is then allowed to proceed by inward diffusion of surface Mn dopants while incorporating more Mn^2+^ ions onto the particle surface (Fig. 5e, step (iii)). The inward-diffused Mn^2+^ ions start to produce internal [MnCl_6_]^4−^ octahedral units and connect with the neighboring [BiCl_6_]^3−^ octahedra, forming the Cs_4_MnBi_2_Cl_12_ LDP motif that coexists with the 0D structure in individual NCs. Upon further increasing the Mn concentration, the 0D perovskite motif was gradually replaced by the 2D LDP structure driven by the latter's enhanced structural stability with balanced charge.^87^ Finally, when the Mn concentration reaches the stoichiometry ratio of [Mn]/[Bi] = 50%, the final Cs_4_MnBi_2_Cl_12_ LDP NCs are obtained (Fig. 5e, step (iv)). The overall transformation reaction can be expressed by the following chemical equations:

5 Cs_3_BiCl_6_ + *x*Mn^2+^ → Cs_3_BiCl_6_:*x*Mn^2+^

6 3Cs_3_BiCl_6_:*x*Mn^2+^ + (1 − 2*x*)Mn^2+^ → Cs_3_BiCl_6_:*x*Mn^2+^·Cs_4_MnBi_2_Cl_12_ + 2Cs^+^

7 2Cs_3_BiCl_6_:*x*Mn^2+^·Cs_4_MnBi_2_Cl_12_ + (1 − 2*x*)Mn^2+^ → 3Cs_4_MnBi_2_Cl_12_ + 2Cs^+^

## Conclusions

In conclusion, we report a facile synthetic strategy towards the fabrication of lead-free Bi-based perovskite NCs based on a post-synthetic transformation reaction of the Cs_3_BiX_6_ (X = Cl, Br) 0D perovskite NCs under mild conditions. Both high-quality Cs_2_AgBiX_6_ 3D DP NCs and Cs_4_M(ii)Bi_2_Cl_12_ (M(ii) = Cd, Mn) 2D LDP NCs can be obtained using this method, resulting in LFHP NCs with uniform size and comparable optical properties to the ones obtained from conventional hot-injection syntheses. The evolution of optical properties and NC structure was investigated, based on which we propose an intra-particle transformation mechanism involving cation-alloyed intermediates. The slow reaction kinetics and stop-on-demand feature of the transformation reaction facilitate the synthesis of Cs_2_(Cs_1−*x*_Ag_*x*_)BiCl_6_ intermediate DP NCs which possess unique STE emission. We anticipate that this post-synthetic NC transformation method can be further expanded to other perovskite and/or even non-perovskite systems. Our study presented here demonstrates a low-energy-input and controllable pathway towards the production of high-quality LFHP NCs post synthesis and paves the road for future exploration of novel perovskite and perovskite-analogue materials and understanding their composition- and structure-related properties.

## Data availability

All the relevant data discussed in the manuscript are provided within the article and in the ESI.†

## Author contributions

H. Y. and O. C. conceived and designed the experiments. H. Y. performed NC synthesis and the transformation reaction. T. C. performed DP NC synthesis and XPS measurements. H. Y. and L. D. performed optical spectra measurements. H. Y. performed XRD, TEM, Raman, and EPR measurements. O. C. supervised the entire projects. The manuscript was written through the contributions of all authors. All authors have given approval to the final version.

## Conflicts of interest

There are no conflicts to declare.

## Supplementary Material

SC-013-D2SC00574C-s001

## References

[cit1] Dey A., Ye J., De A., Debroye E., Ha S. K., Bladt E., Kshirsagar A. S., Wang Z., Yin J., Wang Y., Quan L. N., Yan F., Gao M., Li X., Shamsi J., Debnath T., Cao M., Scheel M. A., Kumar S., Steele J. A., Gerhard M., Chouhan L., Xu K., Wu X.-g., Li Y., Zhang Y., Dutta A., Han C., Vincon I., Rogach A. L., Nag A., Samanta A., Korgel B. A., Shih C.-J., Gamelin D. R., Son D. H., Zeng H., Zhong H., Sun H., Demir H. V., Scheblykin I. G., Mora-Seró I., Stolarczyk J. K., Zhang J. Z., Feldmann J., Hofkens J., Luther J. M., Pérez-Prieto J., Li L., Manna L., Bodnarchuk M. I., Kovalenko M. V., Roeffaers M. B. J., Pradhan N., Mohammed O. F., Bakr O. M., Yang P., Müller-Buschbaum P., Kamat P. V., Bao Q., Zhang Q., Krahne R., Galian R. E., Stranks S. D., Bals S., Biju V., Tisdale W. A., Yan Y., Hoye R. L. Z., Polavarapu L. (2021). ACS Nano.

[cit2] Fan Q., Biesold-McGee G. V., Ma J., Xu Q., Pan S., Peng J., Lin Z. (2020). Angew. Chem., Int. Ed..

[cit3] Wang Y., Song L., Chen Y., Huang W. (2020). ACS Photonics.

[cit4] Ke W., Kanatzidis M. G. (2019). Nat. Commun..

[cit5] Infante I., Manna L. (2021). Nano Lett..

[cit6] Khalfin S., Bekenstein Y. (2019). Nanoscale.

[cit7] Xiao Z., Song Z., Yan Y. (2019). Adv. Mater..

[cit8] Yang H., Zhang Y., Hills-Kimball K., Zhou Y., Chen O. (2018). Sustainable Energy Fuels.

[cit9] Liu Y., Nag A., Manna L., Xia Z. (2021). Angew. Chem., Int. Ed..

[cit10] Slavney A. H., Hu T., Lindenberg A. M., Karunadasa H. I. (2016). J. Am. Chem. Soc..

[cit11] Volonakis G., Haghighirad A. A., Milot R. L., Sio W. H., Filip M. R., Wenger B., Johnston M. B., Herz L. M., Snaith H. J., Giustino F. (2017). J. Phys. Chem. Lett..

[cit12] Pan W., Wu H., Luo J., Deng Z., Ge C., Chen C., Jiang X., Yin W.-J., Niu G., Zhu L., Yin L., Zhou Y., Xie Q., Ke X., Sui M., Tang J. (2017). Nat. Photonics.

[cit13] Vargas B., Ramos E., Pérez-Gutiérrez E., Alonso J. C., Solis-Ibarra D. (2017). J. Am. Chem. Soc..

[cit14] Wei J.-H., Liao J.-F., Wang X.-D., Zhou L., Jiang Y., Kuang D.-B. (2020). Matter.

[cit15] Connor B. A., Leppert L., Smith M. D., Neaton J. B., Karunadasa H. I. (2018). J. Am. Chem. Soc..

[cit16] Chen N., Cai T., Li W., Hills-Kimball K., Yang H., Que M., Nagaoka Y., Liu Z., Yang D., Dong A., Xu C.-Y., Zia R., Chen O. (2019). ACS Appl. Mater. Interfaces.

[cit17] Yao M.-M., Wang L., Yao J.-S., Wang K.-H., Chen C., Zhu B.-S., Yang J.-N., Wang J.-J., Xu W.-P., Zhang Q., Yao H.-B. (2020). Adv. Opt. Mater..

[cit18] Tang H., Xu Y., Hu X., Hu Q., Chen T., Jiang W., Wang L., Jiang W. (2021). Adv. Sci..

[cit19] Zhu X., Bian L., Fu H., Wang L., Zou B., Dai Q., Zhang J., Zhong H. (2020). Light: Sci. Appl..

[cit20] Cong M., Zhang Q., Yang B., Chen J., Xiao J., Zheng D., Zheng T., Zhang R., Qing G., Zhang C., Han K.-l. (2021). Nano Lett..

[cit21] Luo J., Wang X., Li S., Liu J., Guo Y., Niu G., Yao L., Fu Y., Gao L., Dong Q., Zhao C., Leng M., Ma F., Liang W., Wang L., Jin S., Han J., Zhang L., Etheridge J., Wang J., Yan Y., Sargent E. H., Tang J. (2018). Nature.

[cit22] Han P., Zhang X., Luo C., Zhou W., Yang S., Zhao J., Deng W., Han K. (2020). ACS Cent. Sci..

[cit23] Manna D., Das T. K., Yella A. (2019). Chem. Mater..

[cit24] Liu S., Yang B., Chen J., Wei D., Zheng D., Kong Q., Deng W., Han K. (2020). Angew. Chem., Int. Ed..

[cit25] Abfalterer A., Shamsi J., Kubicki D. J., Savory C. N., Xiao J., Divitini G., Li W., Macpherson S., Gałkowski K., MacManus-Driscoll J. L., Scanlon D. O., Stranks S. D. (2020). ACS Mater. Lett..

[cit26] Tan L., Wang W., Li Q., Luo Z., Zou C., Tang M., Zhang L., He J., Quan Z. (2020). Chem. Commun..

[cit27] Cai T., Shi W., Hwang S., Kobbekaduwa K., Nagaoka Y., Yang H., Hills-Kimball K., Zhu H., Wang J., Wang Z., Liu Y., Su D., Gao J., Chen O. (2020). J. Am. Chem. Soc..

[cit28] Cai T., Shi W., Gosztola D. J., Kobbekaduwa K., Yang H., Jin N., Nagaoka Y., Dube L., Schneider J., Hwang S., Gao J., Ma X., Chen O. (2021). Matter.

[cit29] P A. P., Joshi M., Verma D., Jadhav S., Choudhury A. R., Jana D. (2021). ACS Appl. Nano Mater..

[cit30] Yang H., Shi W., Cai T., Hills-Kimball K., Liu Z., Dube L., Chen O. (2020). Nanoscale.

[cit31] Liu Z., Yang H., Wang J., Yuan Y., Hills-Kimball K., Cai T., Wang P., Tang A., Chen O. (2021). Nano Lett..

[cit32] Zhu D., Zito J., Pinchetti V., Dang Z., Olivati A., Pasquale L., Tang A., Zaffalon M. L., Meinardi F., Infante I., De Trizio L., Manna L., Brovelli S. (2020). ACS Energy Lett..

[cit33] Bekenstein Y., Dahl J. C., Huang J., Osowiecki W. T., Swabeck J. K., Chan E. M., Yang P., Alivisatos A. P. (2018). Nano Lett..

[cit34] Creutz S. E., Liu H., Kaiser M. E., Li X., Gamelin D. R. (2019). Chem. Mater..

[cit35] Levy S., Khalfin S., Pavlopoulos N. G., Kauffmann Y., Atiya G., Shaek S., Dror S., Shechter R., Bekenstein Y. (2021). Chem. Mater..

[cit36] Akkerman Q. A., D'Innocenzo V., Accornero S., Scarpellini A., Petrozza A., Prato M., Manna L. (2015). J. Am. Chem. Soc..

[cit37] Nedelcu G., Protesescu L., Yakunin S., Bodnarchuk M. I., Grotevent M. J., Kovalenko M. V. (2015). Nano Lett..

[cit38] Li G. P., Wang H., Zhu Z. F., Chang Y. J., Zhang T., Song Z. H., Jiang Y. (2016). Chem. Commun..

[cit39] Creutz S. E., Crites E. N., De Siena M. C., Gamelin D. R. (2018). Nano Lett..

[cit40] Lou Y., Fang M., Chen J., Zhao Y. (2018). Chem. Commun..

[cit41] Que M., Dai Z., Yang H., Zhu H., Zong Y., Que W., Padture N. P., Zhou Y., Chen O. (2019). ACS Energy Lett..

[cit42] Saidaminov M. I., Mohammed O. F., Bakr O. M. (2017). ACS Energy Lett..

[cit43] Lin H., Zhou C., Tian Y., Siegrist T., Ma B. (2018). ACS Energy Lett..

[cit44] Akkerman Q. A., Park S., Radicchi E., Nunzi F., Mosconi E., De Angelis F., Brescia R., Rastogi P., Prato M., Manna L. (2017). Nano Lett..

[cit45] Palazon F., Almeida G., Akkerman Q. A., De Trizio L., Dang Z., Prato M., Manna L. (2017). Chem. Mater..

[cit46] Palazon F., Urso C., De Trizio L., Akkerman Q., Marras S., Locardi F., Nelli I., Ferretti M., Prato M., Manna L. (2017). ACS Energy Lett..

[cit47] Hu H., Wu L., Tan Y., Zhong Q., Chen M., Qiu Y., Yang D., Sun B., Zhang Q., Yin Y. (2018). J. Am. Chem. Soc..

[cit48] Li Y., Huang H., Xiong Y., Kershaw S. V., Rogach A. L. (2018). CrystEngComm.

[cit49] Yu X., Wu L., Hu H., Chen M., Tan Y., Yang D., Pan Q., Zhong Q., Supasai T., Zhang Q. (2018). Langmuir.

[cit50] Yang H., Cai T., Liu E., Hills-Kimball K., Gao J., Chen O. (2020). Nano Res..

[cit51] Yang H., Cai T., Dube L., Hills-Kimball K., Chen O. (2021). Cryst. Growth Des..

[cit52] Sun X., Shi X., Zhang W., Xu B., Gao Z., Wang Z., Wang X., Meng X. (2021). Nanoscale.

[cit53] Huisman B. A. H., Palazon F., Bolink H. J. (2021). Inorg. Chem..

[cit54] Palazon F., Akkerman Q. A., Prato M., Manna L. (2016). ACS Nano.

[cit55] Billstrand B., Bian K., Karler C., Ye D., Hwang A., Fan H. (2018). MRS Adv..

[cit56] Hills-Kimball K., Yang H., Cai T., Wang J., Chen O. (2021). Adv. Sci..

[cit57] Baranov D., Caputo G., Goldoni L., Dang Z., Scarfiello R., De Trizio L., Portone A., Fabbri F., Camposeo A., Pisignano D., Manna L. (2020). Chem. Sci..

[cit58] Wu L., Hu H., Xu Y., Jiang S., Chen M., Zhong Q., Yang D., Liu Q., Zhao Y., Sun B., Zhang Q., Yin Y. (2017). Nano Lett..

[cit59] Ren J., Zhou X., Wang Y. (2020). Nano Res..

[cit60] Yin J., Maity P., De Bastiani M., Dursun I., Bakr O. M., Brédas J.-L., Mohammed O. F. (2017). Sci. Adv..

[cit61] Tang Y., Liang M., Chang B., Sun H., Zheng K., Pullerits T., Chi Q. (2019). J. Mater. Chem. C.

[cit62] Schmitz A., Schaberg L. L., Sirotinskaya S., Pantaler M., Lupascu D. C., Benson N., Bacher G. (2020). ACS Energy Lett..

[cit63] Shannon R. (1976). Acta Crystallogr., Sect. A: Cryst. Phys., Diffr., Theor. Gen. Crystallogr..

[cit64] Lee S., Park J. H., Lee B. R., Jung E. D., Yu J. C., Di Nuzzo D., Friend R. H., Song M. H. (2017). J. Phys. Chem. Lett..

[cit65] Smit W. M. A., Dirksen G. J., Stufkens D. J. (1990). J. Phys. Chem. Solids.

[cit66] Zelewski S. J., Urban J. M., Surrente A., Maude D. K., Kuc A., Schade L., Johnson R. D., Dollmann M., Nayak P. K., Snaith H. J., Radaelli P., Kudrawiec R., Nicholas R. J., Plochocka P., Baranowski M. (2019). J. Mater. Chem. C.

[cit67] Shi W., Cai T., Wang Z., Chen O. (2020). J. Chem. Phys..

[cit68] Travis W., Glover E. N. K., Bronstein H., Scanlon D. O., Palgrave R. G. (2016). Chem. Sci..

[cit69] Xiao Z., Meng W., Wang J., Mitzi D. B., Yan Y. (2017). Mater. Horiz..

[cit70] Ke B., Zeng R., Zhao Z., Wei Q., Xue X., Bai K., Cai C., Zhou W., Xia Z., Zou B. (2020). J. Phys. Chem. Lett..

[cit71] Lamba R. S., Basera P., Bhattacharya S., Sapra S. (2019). J. Phys. Chem. Lett..

[cit72] Benin B. M., Dirin D. N., Morad V., Wörle M., Yakunin S., Rainò G., Nazarenko O., Fischer M., Infante I., Kovalenko M. V. (2018). Angew. Chem., Int. Ed..

[cit73] Locardi F., Sartori E., Buha J., Zito J., Prato M., Pinchetti V., Zaffalon M. L., Ferretti M., Brovelli S., Infante I., De Trizio L., Manna L. (2019). ACS Energy Lett..

[cit74] Cortecchia D., Neutzner S., Srimath Kandada A. R., Mosconi E., Meggiolaro D., De Angelis F., Soci C., Petrozza A. (2017). J. Am. Chem. Soc..

[cit75] Stranks S. D., Burlakov V. M., Leijtens T., Ball J. M., Goriely A., Snaith H. J. (2014). Phys. Rev. Appl..

[cit76] Steele J. A., Puech P., Keshavarz M., Yang R., Banerjee S., Debroye E., Kim C. W., Yuan H., Heo N. H., Vanacken J., Walsh A., Hofkens J., Roeffaers M. B. J. (2018). ACS Nano.

[cit77] Zhang L., Fang Y., Sui L., Yan J., Wang K., Yuan K., Mao W. L., Zou B. (2019). ACS Energy Lett..

[cit78] Liu W., Lin Q., Li H., Wu K., Robel I., Pietryga J. M., Klimov V. I. (2016). J. Am. Chem. Soc..

[cit79] Cai T., Wang J., Li W., Hills-Kimball K., Yang H., Nagaoka Y., Yuan Y., Zia R., Chen O. (2020). Adv. Sci..

[cit80] Vargas B., Reyes-Castillo D. T., Coutino-Gonzalez E., Sánchez-Aké C., Ramos C., Falcony C., Solis-Ibarra D. (2020). Chem. Mater..

[cit81] Hills-Kimball K., Pérez M. J., Nagaoka Y., Cai T., Yang H., Davis A. H., Zheng W., Chen O. (2020). Chem. Mater..

[cit82] Xu J., Xu C., Liu J.-B., Bellaiche L., Xiang H., Liu B.-X., Huang B. (2019). npj Comput. Mater..

[cit83] Tsay F. D., Helmholz L. (1969). J. Chem. Phys..

[cit84] Davis A. H., Li S., Lin H., Chu C., Franck J. M., Leem G., Maye M. M., Zheng W. (2021). J. Mater. Chem. C.

[cit85] Liu Y., Zhang J., Han B., Wang X., Wang Z., Xue C., Bian G., Hu D., Zhou R., Li D.-S., Wang Z., Ouyang Z., Li M., Wu T. (2020). J. Am. Chem. Soc..

[cit86] Abhyankar N., Bertaina S., Dalal N. S. (2015). J. Phys. Chem. C.

[cit87] Lin Y.-P., Hu S., Xia B., Fan K.-Q., Gong L.-K., Kong J.-T., Huang X.-Y., Xiao Z., Du K.-Z. (2019). J. Phys. Chem. Lett..

