# Supplementary material for: Synthesis of double perovskite and quadruple perovskite nanocrystals through post-synthetic transformation reactions
Source: Chem Sci. 2022 Mar 30;13(17):4874–83. doi: 10.1039/d2sc00574c (PMC9067587; doi:10.1039/d2sc00574c)
Supplement: SC-013-D2SC00574C-s001 [file SC-013-D2SC00574C-s001.pdf]

Electronic Supplementary Material (ESI) for Chemical Science.
This journal is © The Royal Society of Chemistry 2022

Synthesis of Double Perovskite and Quadruple Perovskite Nanocrystals through Post-Synthetic Transformation Reactions

*Hanjun Yang, Tong Cai, Lacie Dube, and Ou Chen**

Department of Chemistry, Brown University, 324 Brook St., Providence, Rhode Island 02912, United States

E-mail: ouchen@brown.edu

1. Chemicals

Cesium carbonate (Cs_2CO_3, 99.9%), cesium acetate (Cs(OAc), 99.9%), bismuth acetate ($Bi(OAc)_3$, >99.99%), manganese chloride ($MnCl_2$, >99%), manganese acetate (Mn(OAc), anhydrous, 98%), cadmium chloride ($CdCl_2$, >99.99%, trace metal basis), silver nitrate ($AgNO_3$, 99.9999%, trace metal basis), silver acetate (Ag(OAc), 99.99%), benzoyl bromide (97%), chlorotrimethylsilane (TMS-Cl, >99%), bromotrimethylsilane (TMS-Br, 97%), 1-octadecene (ODE, technical grade, 90%), oleylamine (OAm, technical grade, 70%), and oleic acid (OA, technical grade, 90%) were obtained from Aldrich. Cadmium acetate dihydrate ($Cd(OAc)_2 \cdot 2H_2O$, 99.999%) and benzoyl chloride (99%) were obtained from Alfa Aesar. Hexane, toluene, acetonitrile, and ethyl acetate were obtained from Fisher. All chemicals were used as received without further purification.

2. Experimental Procedure

Synthesis of Cs_3BiX_6 nanocrystals (NCs)

The synthesis procedure of Cs_3BiX_6 NCs was based on a previous report method by our group with minor modification.[1] Bismuth acetate (200 mg, 0.52 mmol) and cesium carbonate (80 mg, 0.25 mmol) were mixed with ODE (20 mL), OA (1 mL), and OAm (3 mL) and degassed at 90°C under vacuum. Afterward, the mixture was heated to 160°C when a mixture of 0.8 mL benzoyl halide (3.4 mmol) and 0.8 mL ODE was rapidly injected. The solution was immediately submerged under an ice-water bath and cooled down to room temperature. The resulting NCs were separated

from the solution by centrifuge at 6000 rpm for 5 min, then purified twice by toluene and ethyl acetate. The resulting NCs were dispersed in toluene or hexane for further use.

Post-synthetic transformation of Cs_3BiX_6 NCs to double perovskite (DP) NCs

In a typical reaction, 5 mL of Cs_3BiX_6 NC hexane dispersion (2 mg/mL) with 5 µL OA and 5 µL OAm was heated to 50°C under constant stirring. Certain amounts of 10 mg/mL $AgNO_3$ solution in acetonitrile were added slowly to the Cs_3BiX_6 dispersion until the ratio of Ag:Bi reached 1:1. The stoichiometric ratio of Ag and Bi was determined based on the mass of starting 0D NCs and the added volume of $AgNO_3$ solution. After each addition, the solution was allowed to react for at least 30 minutes until no further change in the absorption profile was observed. After the reaction was completed, the reaction solution was centrifuged at 3000 rpm for 3 minutes, and the clear solution containing the resulting NCs was collected.

Post-synthetic transformation of Cs_3BiCl_6 NCs to layered double perovskite (LDP) NCs

In a typical reaction, 5 mL of Cs_3BiCl_6 NC toluene dispersion (2 mg/mL) was heated to 50°C under constant stirring. 10 mg $MnCl_2$ or $CdCl_2$ powder was mixed with the Cs_3BiCl_6 solution, and the reaction was allowed to last for 24 hours. The final reaction solution was centrifuged at 3000 rpm for 3 minutes, and the clear solution containing the resulting NCs was collected.

Direct synthesis of DP NCs

The synthesis of $Cs_2AgBiCl_6$ NCs was based on a previous report.[2] Cs(OAc) (68 mg, 0.35 mmol), Ag(OAc) (42 mg, 0.25 mmol) and Bi(OAc) (97 mg, 0.25 mmol) were mixed in ODE (5 mL), oleic acid (1.25 g) and oleylamine (0.25 g). The mixture was degassed at 110 °C under vacuum for 45 min. Afterward, the mixture was heated to 140°C when TMS-X (1.4 mmol, 0.2 mL for either TMS-Cl or TMS-Br) were swiftly injected. The reaction mixture was immediately submerged under an ice-water bath and cooled down to room temperature. The resulting solution were mixed with 5 mL of toluene and resuspended. The NCs were separated from the solution by centrifuge at 6000 rpm for 5 min and the precipitates were discarded. The NCs were then purified once more by ethyl acetate. The resulting NCs were dispersed in toluene for further use.

Direct synthesis of LDP NCs

The synthesis of $Cs_4MnBi_2Cl_{12}$ NCs was based on a previous report method by our group.[3] Cs_2CO_3 (40 mg, 0.12 mmol), $Bi(OAc)_3$ (100 mg, 0.26 mmol) and metal acetate ($Cd(OAc)_2{\cdot}2H_2O$ (69.0 mg, 0.26 mmol) or $Mn(OAc)_2{\cdot}4H_2O$ (63.5 mg, 0.26 mmol)) were mixed in ODE (10 mL). OA(1.5 mL) and OAm (0.5 mL) were added to the ODE solution. The mixture was degassed at 70 °C for 1 hour, then heated at 100 °C for an additional 30 minutes. The mixture was then heated in nitrogen atmosphere to 170 °C. Upon reaching 170 °C, a mixture of 0.4 mL benzoyl halide (1.7 mmol) and 0.4 mL ODE was rapidly injected, which initiated the nucleation and growth of NCs. The mixture was allowed to react at 170 °C for 30 seconds, before being cooled in an ice-water bath. The NCs were separated from ODE by adding isopropanol, then centrifuged at 8000 rpm for 10 minutes. The precipitate that contained the NCs was then redispersed in toluene.

3. Characterizations

X-ray diffraction (XRD) measurements

Powder XRD patterns were obtained on a Bruker D8 Discovery 2D X-ray Diffractometer equipped with a Vantec 500 2D area detector working on Cu Kα radiation. NC samples were drop-cast on a glass slide under gentle heating to evaporate the solvent. The crystalline domain size is calculated using Scherrer's formula,

$$S = \frac{K\lambda}{\beta cos\theta}$$

where S is the calculated crystalline domain size, K is the shape factor (0.9), λ is the X-ray wavelength (0.154 nm), β is the full-width-at-half-maximum (FWHM) of diffraction peak, and θ is the diffraction peak position.

X-ray photoelectron spectroscopy (XPS) measurements

XPS spectra were measured on a Thermo Scientific KAlpha+ instrument operating on Al Kα=1486.6 eV radiation with a spot size of ~ 200 μm. Samples dispersions were drop-cast on silicon wafers and allowed to evaporate with gentle heating. All XPS signals were calibrated by setting the adventitious carbon 1s signal to 284.8 eV.

Transmission electron microscopy (TEM) measurements

TEM measurements were carried out using a JEOL-2100F TEM operating at 200kV. Samples were drop-cast on a 300-mesh TEM grid and allowed to dry under ambient conditions. Energy dispersion X-ray spectroscopy (EDS) was measured in TEM mode using Oxford Instrument X-Max 65T EDS probe.

Ultraviolet-visible (UV-Vis) absorptions spectroscopy measurements of NCs

Absorption spectra were measured on an Agilent Cary 8454 UV-Vis Spectrometer. Samples were dispersed in toluene for measurement.

Photoluminescence (PL), PL excitation (PLE) spectroscopy and PL quantum yield (PL QY) measurements

PL and PLE spectra were measured using an Edinburgh Instruments FS5 Spectrofluorometer. NCs were dispersed in toluene and 330 nm UV light was used as the excitation source for all the PL measurements. All PLE measurements were monitored at the PL peak wavelength. PL QYs were determined using a built-in integrating sphere on the Edinburgh Instruments FS5 Spectrofluorometer.

Time-resolved PL (TR-PL) decay measurements of NCs

The TR-PL decay curves were measured using an Edinburgh Instruments FS5 Spectrofluorometer equipped with a 5W microsecond Xenon flashlamp with a repetition rate of 50 Hz. The solution-based samples were dispersed in toluene in a cuvette and excited at 330 nm. The obtained lifetime decays were fitted with bi-exponential or tri-exponential decay functions:

$$I(t) = \sum_k I_k \exp\left(-t/\tau_k\right)$$

Where, $I(t)$ represents the observed PL intensity at the time of t, I_k and τ_k represent the amplitude and lifetime of exponential decay component of k, respectively. Average lifetimes were calculated by the following equation:

$$\tau_{avg} = \frac{\sum_k I_k \tau_k^2}{\sum_k I_k \tau_k}$$

The relative percentage of the decay component i (P_i) was calculated by the following equation:

$$P_i = \frac{I_i \tau_i}{\sum_k I_k \tau_k}$$

Electron paramagnetic resonance (EPR) spectroscopy measurements

EPR spectra were measured on a Bruker EMX Plus EPR Spectrometer. Measurements were taken at room temperature with a 9.86 GHz X-band microwave frequency. NC samples were dissolved in toluene for the measurement.

Raman spectroscopy measurements

Raman spectra were measured using a Witec Alpha 300 confocal Raman microscope excited by 532 nm laser. All samples are drop-cast and evaporated under mild heating on glass slides before measurements. Raman spectra were collected in the 0 ~ 1200 cm^{-1} wavenumber range with a spectral resolution of 1.2 cm^{-1}.

4. Supporting Figures and Tables

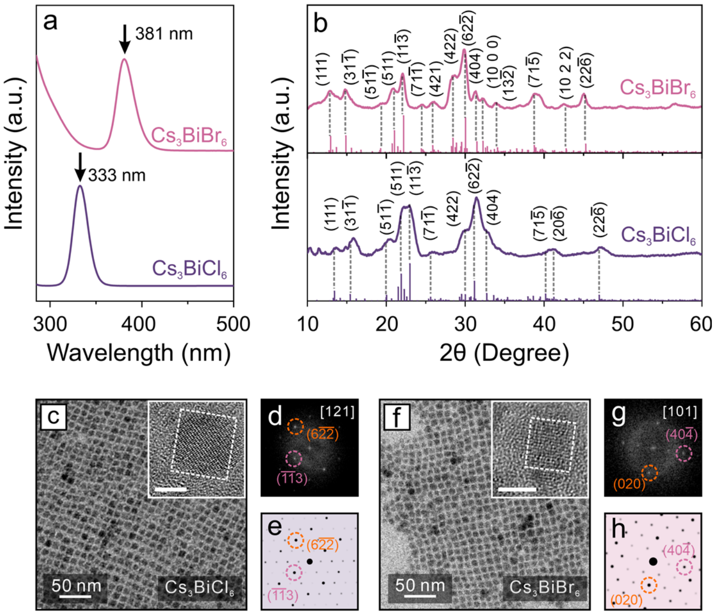

Figure S1. Properties of the Cs_3BiX_6 0D perovskite NCs. (a) The absorption spectra of Cs_3BiCl_6 (bottom) and Cs_3BiBr_6 (top) NCs. (b) XRD patterns of Cs_3BiCl_6 (bottom) and Cs_3BiBr_6 (top) NCs. Standard patterns are marked with bars. The standard XRD pattern of Cs_3BiCl_6 was obtained from Inorganic Crystal Structure Database (ICSD) #201982 and the Cs_3BiBr_6 standard pattern was simulated based on the Cs_3BiCl_6 structure. (c-h). Representative TEM images (c, f), high-resolution (HR) TEM images (c, f: insets, scale bars: 5 nm), the corresponding fast-Fourier transformation (FFT) patterns (d, g) and the simulated electron diffractions (e, h) of the Cs_3BiCl_6 (c-e) and Cs_3BiBr_6 (f-h) 0D perovskite NCs.

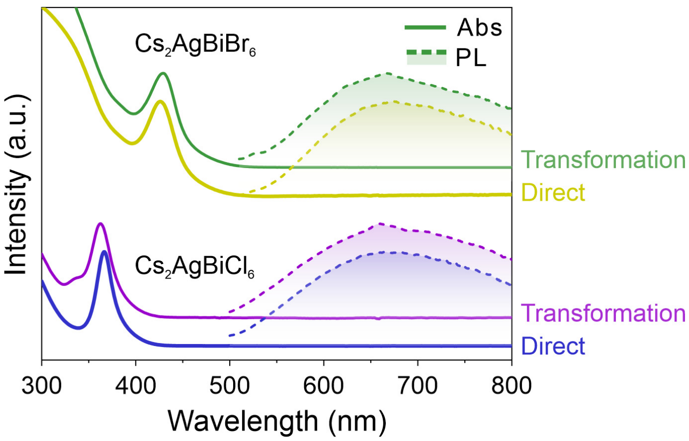

Figure S2. Absorption (Abs, solid lines) and PL (dash lines) spectra of the DP NCs synthesized via the direct synthesis and post-synthesis transformation method.

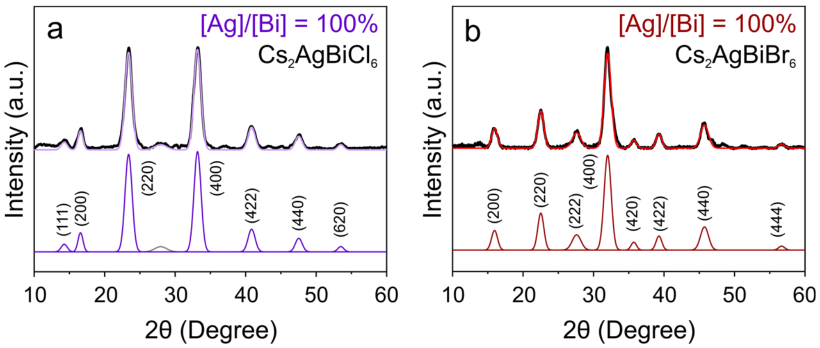

Figure S3 Fitted XRD patterns of the $Cs_2AgBiCl_6$ NCs (a) and $Cs_2AgBiBr_6$ NCs (b) obtained by the DP transformation reactions. All XRD peaks were fitted to gaussian curves. Grey fitted peaks were not used for the fitting of lattice parameters.

Table S1. Fitting parameters of the XRD patterns of the $Cs_2AgBiCl_6$ DP NCs obtained via the transformation reaction.

Index	2θ (°)	FWHM (°)	d (Å)	Miller indices	a (Å)	Crystallite size (nm)
1	14.3	1.03	6.21	(111)	10.8	7.8
2	16.6	0.83	5.35	(200)	10.7	9.7
3	23.4	1.18	3.80	(220)	10.8	6.9
4	33.2	1.18	2.70	(400)	10.8	7.0
5	40.8	1.17	2.21	(422)	10.8	7.2
6	47.5	1.15	1.91	(440)	10.8	7.5
7	53.5	1.00	1.71	(620)	10.8	8.9
Avg.	-	-	-	-	10.8 ± 0.05	7.9 ± 1.0

Table S2. Fitting parameters of the XRD patterns of the $Cs_2AgBiBr_6$ DP NCs obtained via the transformation reaction.

Index	2θ (°)	FWHM (°)	d (Å)	Miller indices	a (Å)	Crystallite size (nm)
1	15.8	1.00	5.63	(200)	11.3	8.0
2	22.3	1.01	3.98	(220)	11.3	8.0
3	27.4	1.47	3.26	(222)	11.3	5.6
4	31.8	1.18	2.81	(400)	11.3	7.0
5	35.5	0.90	2.53	(420)	11.3	9.2
6	39.1	0.96	2.31	(422)	11.3	8.8
7	45.5	1.34	1.99	(440)	11.3	6.4
8	56.5	1.00	1.63	(444)	11.3	9.1
Avg.	-	-	-	-	11.3 ± 0.02	7.8 ± 1.3

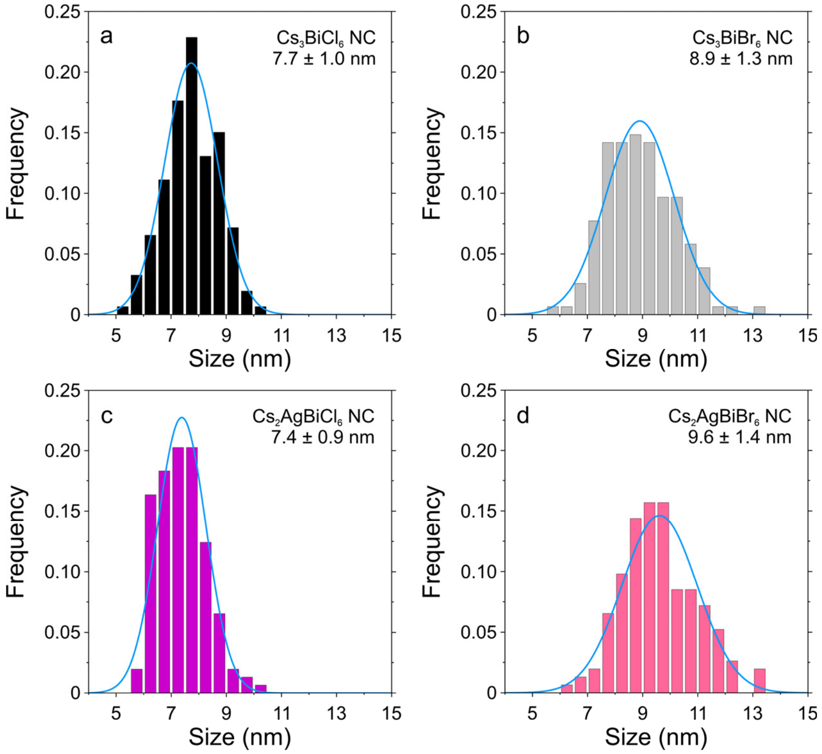

Figure S4. Particle size distribution histograms of Cs_3BiCl_6 NCs (a), Cs_3BiBr_6 NCs (b), $Cs_2AgBiCl_6$ (c) NCs and $Cs_2AgBiBr_6$ NCs (d). More than 150 NCs for samples were used for each plot.

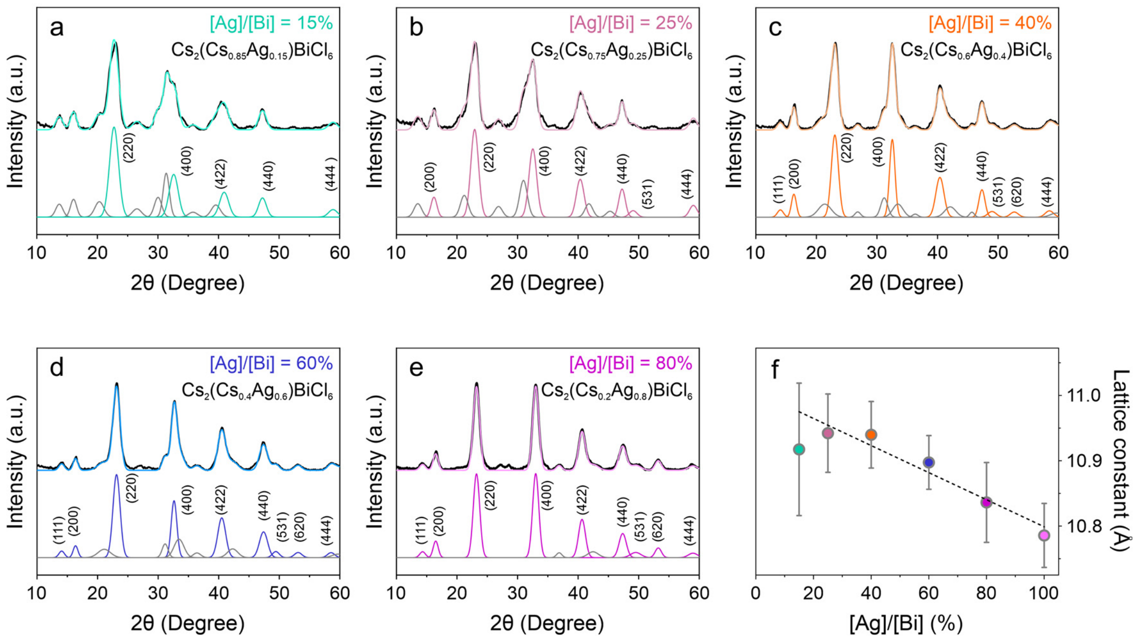

Figure S5. Fitted XRD patterns of the $Cs_2(Cs_{1-x}Ag_x)BiCl_6$ intermediates during the DP transformation reactions. (a-e) [Ag]/[Bi] = 15%, 25%, 40%, 60%, 80%. Gray lines: individual fitted peaks that were not used for the fitting of lattice parameters. All XRD peaks were fitted to gaussian curves. (f) Summary of the lattice constant change while increasing the [Ag]/[Bi] ratio.

Table S3. Fitting parameters of the XRD patterns of the intermediate with [Ag]/[Bi] =15% during the DP transformation reaction from Cs_3BiCl_6 NCs. [Ag]/[Bi] =15%.

Index	2θ (°)	FWHM (°)	d (Å)	Miller indices	a (Å)	Crystallite size (nm)
1	22.8	1.75*	3.91	(220)	11.1	–
2	32.6	1.75*	2.75	(400)	11.0	–
3	40.9	1.75*	2.21	(422)	10.8	–
4	47.3	1.52	1.92	(440)	10.9	5.7
5	58.9	1.75*	1.57	(444)	10.9	–
Avg.	-	-	-	-	10.9 ± 0.1	5.7

*: Values are fixed during fitting

Table S4. Fitting parameters of the XRD patterns of the intermediate with [Ag]/[Bi] = 25% during the DP transformation reaction from Cs_3BiCl_6 NCs.

**Index**	**2θ (°)**	**FWHM (°)**	**d (Å)**	**Miller indices**	**a (Å)**	**Crystallite size (nm)**
1	16.2	1.16	5.47	(200)	11.0	6.9
2	22.9	1.50*	3.88	(220)	11.0	–
3	32.5	1.50*	2.75	(400)	11.0	–
4	40.4	1.50*	2.24	(422)	11.0	–
5	47.3	1.26	1.92	(440)	10.9	6.9
6	49.1	1.50*	1.86	(531)	11.0	–
7	59.0	1.50*	1.57	(444)	10.8	–
Avg.	-	-	-	-	10.9 ± 0.1	6.9 ± 0.1

*: Values are fixed during fitting

Table S5. Fitting parameters of the XRD patterns of the intermediate with [Ag]/[Bi] = 40% during the DP transformation reaction from Cs_3BiCl_6 NCs.

Index	2θ (°)	FWHM (°)	d (Å)	Miller indices	a (Å)	Crystallite size (nm)
1	14.1	1.07	6.30	(111)	10.9	6.8
2	16.3	0.92	5.44	(200)	10.9	8.4
3	23.1	1.39	3.86	(220)	10.9	5.9
4	32.5	1.10	2.75	(400)	11.0	7.8
5	40.4	1.53	2.23	(422)	10.9	5.4
6	47.3	1.59	1.92	(440)	10.9	7.3
7	49.0	1.33	1.86	(531)	11.0	5.3
8	52.7	1.43	1.74	(620)	11.0	5.6
9	58.5	1.41	1.58	(444)	10.9	5.5
Avg.	-	-	-	-	10.9 ± 0.05	6.5 ± 1.2

Table S6. Fitting parameters of the XRD patterns of the intermediate with [Ag]/[Bi] = 60% during the DP transformation reaction from Cs_3BiCl_6 NCs.

Index	2θ (°)	FWHM (°)	d (Å)	Miller indices	a (Å)	Crystallite size (nm)
1	14.1	1.02	6.29	(111)	10.9	7.9
2	16.4	0.91	5.42	(200)	10.8	8.9
3	23.1	1.38	3.85	(220)	10.9	5.9
4	32.6	1.10	2.75	(400)	11.0	7.5
5	40.5	1.53	2.23	(422)	10.9	5.5
6	47.4	1.59	1.92	(440)	10.9	5.5
7	49.5	1.32	1.84	(531)	10.9	6.6
8	53.1	1.41	1.72	(620)	10.9	6.3
9	58.6	1.54	1.58	(444)	10.9	5.9
Avg.	-	-	-	-	10.9 ± 0.04	6.7 ± 1.2

Table S7. Fitting parameters of the XRD patterns of the intermediate with [Ag]/[Bi] = 80% during the DP transformation reaction from Cs_3BiCl_6 NCs.

**Index**	**2θ (°)**	**FWHM (°)**	**d (Å)**	**Miller indices**	**a (Å)**	**Crystallite size (nm)**
1	14.3	1.10	6.19	(111)	10.7	7.3
2	16.5	0.99	5.38	(200)	10.8	8.2
3	23.2	1.25	3.83	(220)	10.8	6.5
4	33.0	1.21	2.71	(400)	10.9	6.9
5	40.6	1.33	2.22	(422)	10.9	6.4
6	47.4	1.41	1.92	(440)	10.9	6.2
7	49.5	2.02	1.84	(531)	10.9	4.3
8	53.2	1.21	1.72	(620)	10.9	7.3
9	59.0	1.78	1.57	(444)	10.9	5.1
Avg.	-	-	-	-	10.8 ± 0.06	6.5 ± 1.2

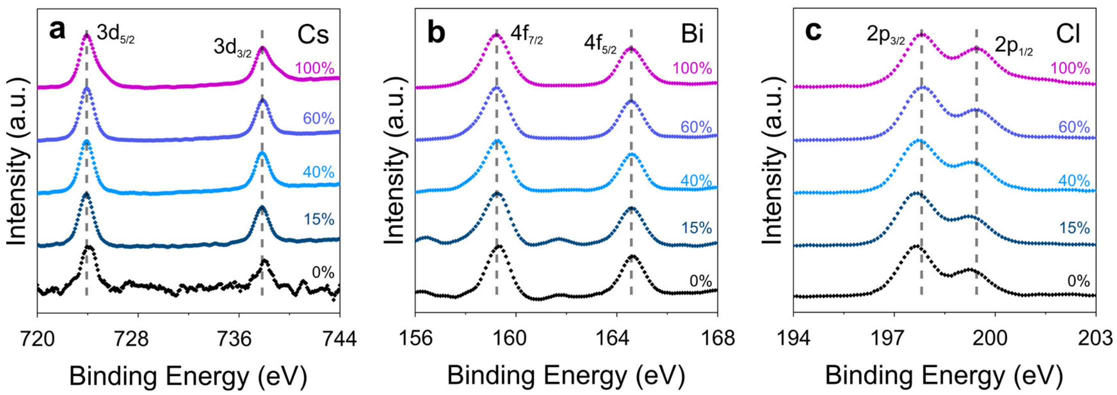

Figure S6. XPS spectra of the $Cs_2(Cs_{1-x}Ag_x)BiCl_6$ intermediate NCs during the DP transformation reaction. (a) Cs 3d, (b) Bi 4f and (c) Cl 2p.

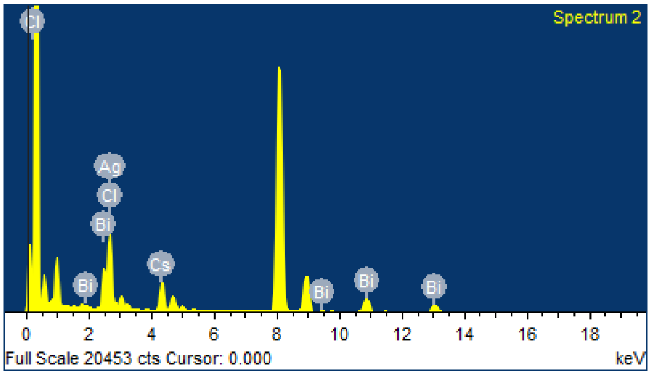

Figure S7. Representative EDS spectrum of the $Cs_2(Cs_{0.6}Ag_{0.4})BiCl_6$ intermediate NCs.

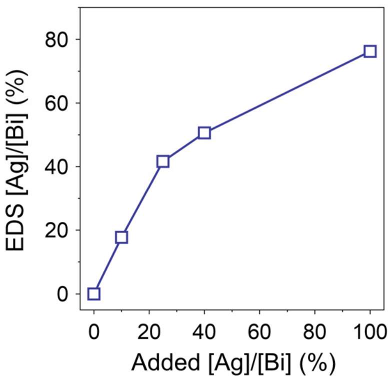

Figure S8. EDS measured [Ag]/[Bi] ratios of the intermediate $Cs_2(Cs_{1-x}Ag_x)BiCl_6$ NCs with different added [Ag]/[Bi] ratios.

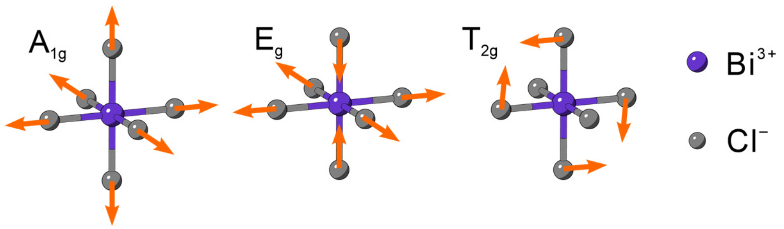

Figure S9. Raman active modes in the $[BiCl_6]^{3-}$ octahedron.

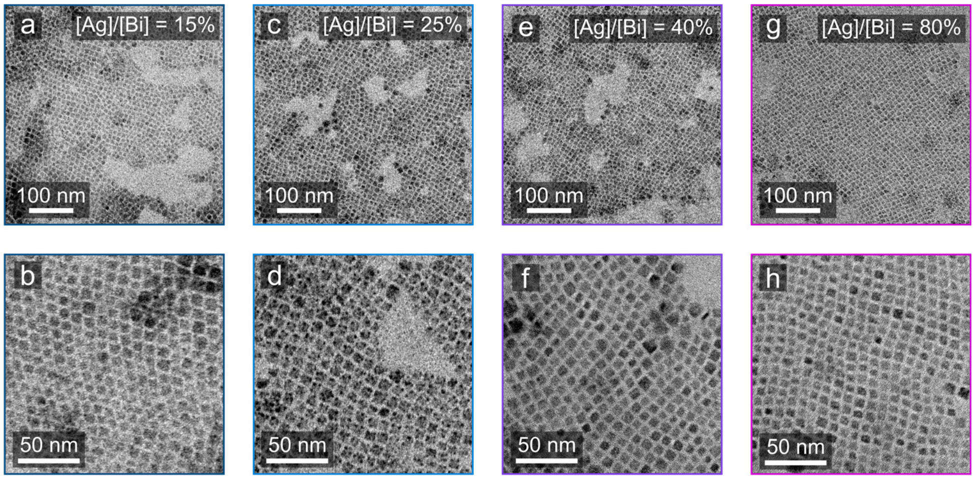

Figure S10. TEM images of the intermediate samples during the transformation from the Cs_3BiCl_6 0D perovskite NCs to $Cs_2AgBiCl_6$ DP NCs. (a, b) [Ag]/[Bi]=15%; (c, d) [Ag]/[Bi]=25%; (e, f) [Ag]/[Bi]=40%; (g, h) [Ag]/[Bi]=80%.

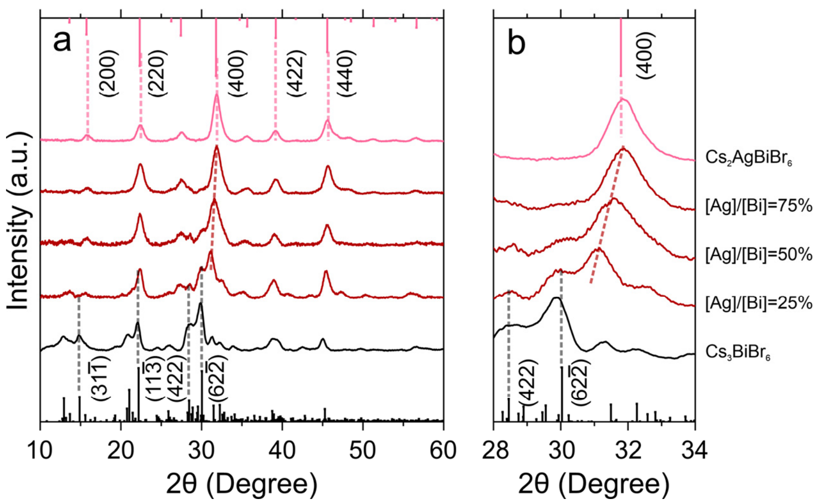

Figure S11. (a) XRD pattern evolution during the transformation reaction from the Cs_3BiBr_6 0D perovskite NCs to $Cs_2AgBiBr_6$ DP NCs. (b) Zoom-in view of the double perovskite (400) peak.

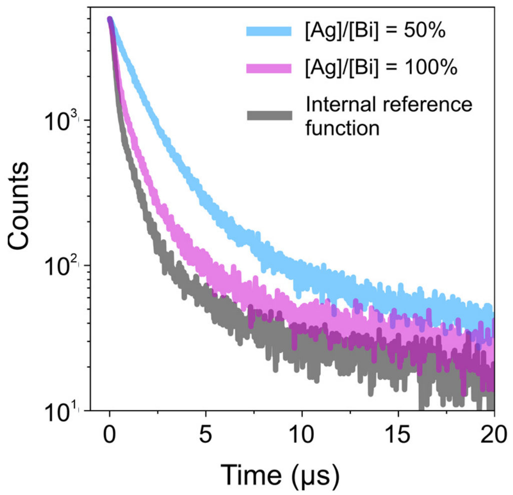

Figure S12. PL lifetime decays for the $Cs_2(Cs_{0.5}Ag_{0.5})BiCl_6$ intermediate NCs ([Ag]/[Bi] = 50%, blue) and the final $Cs_2AgBiCl_6$ DP NCs ([Ag]/[Bi] = 100%, pink) of the transformation reaction. Gray line represents the instrument response decay curve.

Table S8. Biexponential deconvolution fitting parameters of the time-resolved PL spectra of the DP transformation intermediate with [Ag]/[Bi]=50%.

**τ (ns)**	**Rel. percentage (%)**	**τ_{avg} (ns)**
254 ± 5	35.3 ± 0.6	969 ± 9
1359 ± 9	64.7 ± 0.6	

The multi-exponential fitting of [Ag]/[Bi] = 100% was not conducted because it is indistinguishable with the internal reference function.

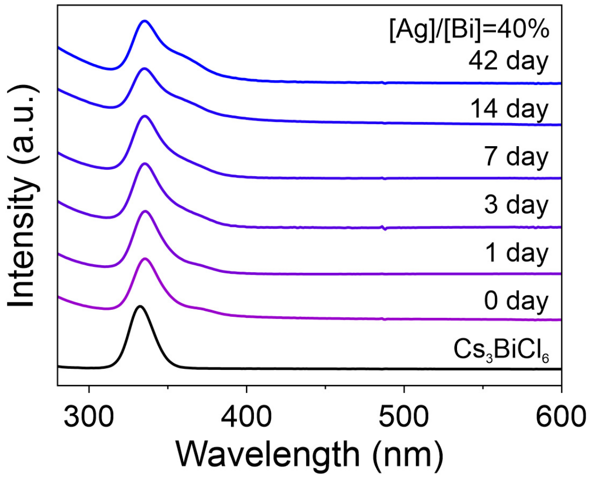

Figure S13. Absorption spectra of the intermediate $Cs_2(Cs_{0.6}Ag_{0.4})BiCl_6$ perovskite NCs taken at different time during the ambient storage.

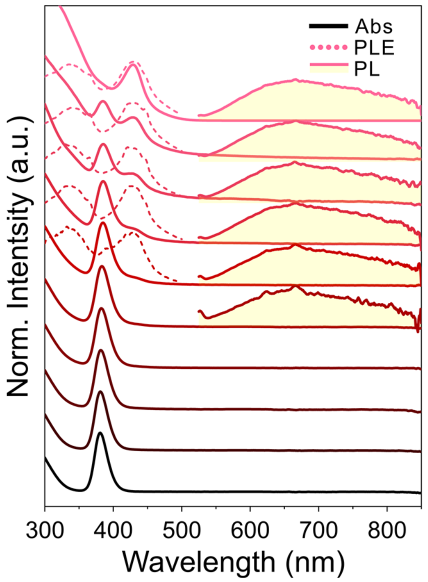

Figure S14. Spectral evolution of the absorption (solid lines), PL (solid lines with shaded area) and PLE (dashed lines) for the transformation reaction of Cs_3BiBr_6 NCs to $Cs_2AgBiBr_6$ DP NCs.

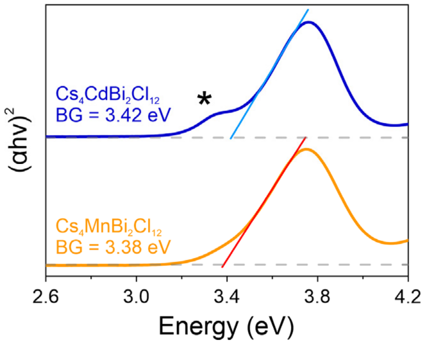

Figure S15. Tauc plot fittings of $Cs_4MnBi_2Cl_{12}$ NCs (orange) and $Cs_4CdBi_2Cl_{12}$ NCs (blue) based on direct band gaps. Asterisk marks an impurity peak, which may originate from the $Cs_3Bi_2Cl_9$ impurity.

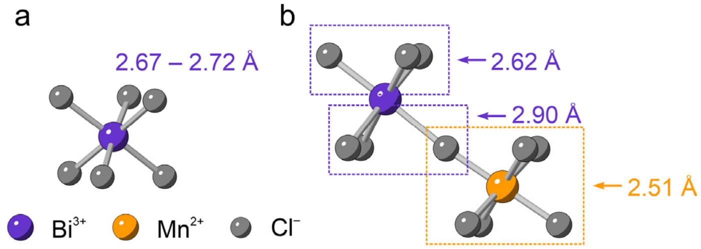

Figure S16. Bond lengths of the Bi-Cl and Mn-Cl bonds in (a) Cs_3BiCl_6 and (b) $Cs_4MnBi_2Cl_{12}$ crystal structures.

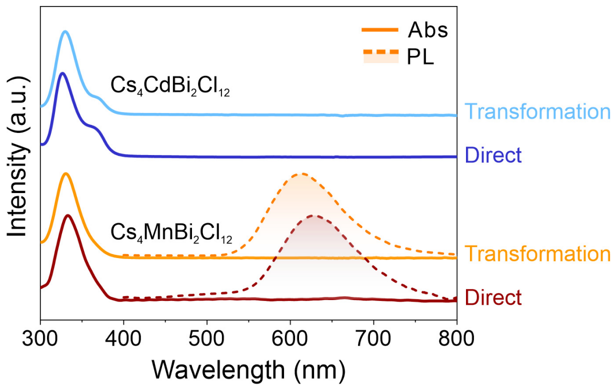

Figure S17. Absorption (Abs, solid lines) and PL (dash lines) spectra of the Cs_4MBiCl_{12} (M = Cd, Mn) LDP NCs synthesized via the direct synthesis and post-synthesis transformation method.

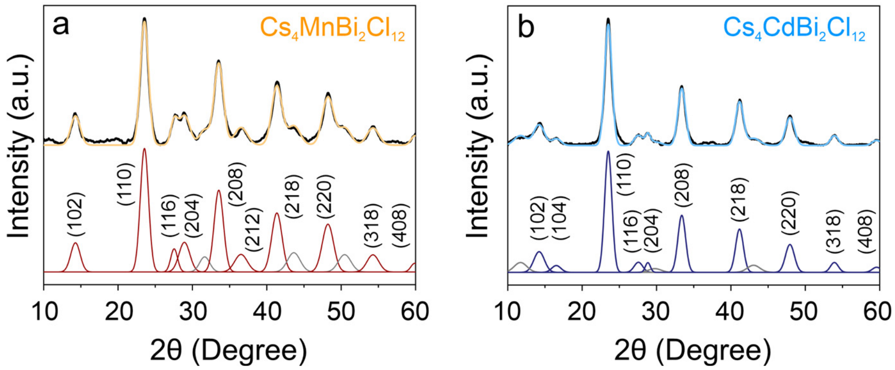

Figure S18. Fitted XRD patterns of the $Cs_4MnBi_2Cl_{12}$ NCs (a) and $Cs_4CdBi_2Cl_{12}$ NCs (b) obtained by the transformation reaction. All XRD peaks were fitted to gaussian curves. Grey fitted peaks were not used for the fitting of lattice parameters.

Table S9. Fitting parameters of the XRD patterns of the $Cs_4MnBi_2Cl_{12}$ LDP NCs obtained via the transformation reaction from Cs_3BiCl_6 NCs.

Index	2θ (°)	FWHM (°)	d (Å)	Miller indices	Crystallite size (nm)
1	14.3	1.49	6.20	(102)	5.4
2	23.6	1.30	3.78	(110)	6.2
3	27.5	1.08	3.24	(116)	7.6
4	28.9	1.72	3.09	(204)	4.8
5	33.5	1.46	2.67	(208)	5.7
6	36.5	2.00*	2.46	(212)	4.2
7	41.4	1.63	2.18	(218)	5.2
8	48.2	1.78	1.89	(220)	4.9
9	50.5	2.00*	1.81	(312)	4.4
10	54.3	1.88	1.69	(318)	4.7
11	59.9	1.20	1.54	(408)	7.6
12	65.3	1.77	1.43	(410)	5.3

*: Values are fixed during fitting

Averages:

a = 7.56 ± 0.01 Å

c = 37.2 ± 0.1 Å

Crystallite size = 5.5 ± 1.1 Å

Table S10. Fitting parameters of the XRD patterns of the $Cs_4CdBi_2Cl_{12}$ LDP NCs obtained via the transformation reaction from Cs_3BiCl_6 NCs.

**Index**	**2θ (°)**	**FWHM (°)**	**d (Å)**	**Miller indices**	**Crystallite size (nm)**
1	14.2	1.67	6.23	(102)	4.8
2	16.5	1.45	5.36	(104)	5.5
3	23.5	1.15	3.78	(110)	7.1
4	27.6	1.35	3.24	(116)	6.1
5	28.8	0.79	3.10	(204)	10.4
6	33.4	1.21	2.68	(208)	6.8
7	41.2	1.23	2.19	(218)	6.9
8	47.9	1.41	1.90	(220)	6.2
9	53.9	1.26	1.70	(318)	7.1
10	59.6	1.38	1.55	(408)	6.6
11	64.9	1.76	1.44	(410)	5.3

Averages:

a = 7.60 ± 0.01 Å

c = 37.2 ± 0.1 Å

Crystallite size = 6.6 ± 1.5 Å

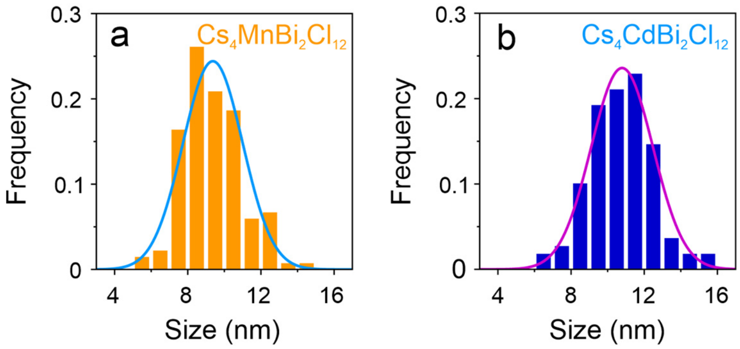

Figure S19. Particle size distribution histograms of the $Cs_4MnBi_2Cl_{12}$ (a) and $Cs_4CdBi_2Cl_{12}$ (b) NCs obtained through the transformation reactions.

Size:

$Cs_4MnBi_2Cl_{12}$ = 9.4 ± 1.6 nm

$Cs_4CdBi_2Cl_{12}$ = 10.8 ± 1.7 nm

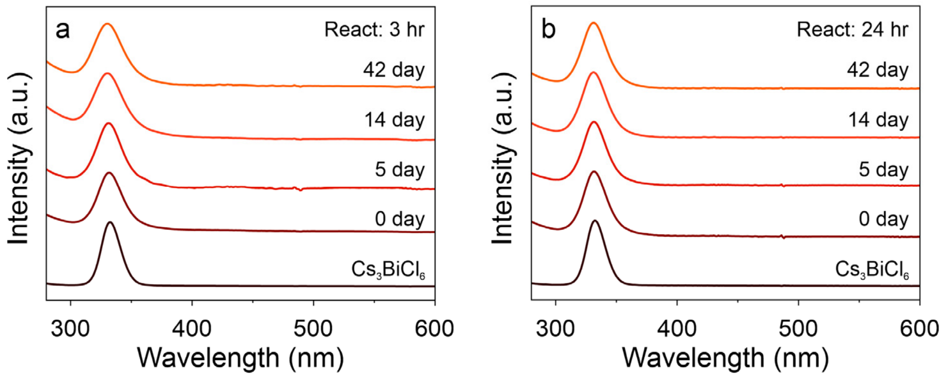

Figure S20. Absorption spectra taken at different times during ambient storage for the intermediate LDP NCs (the sample collected at 3 hr of the LDP transformation reaction) (a), and completely transformed $Cs_4MnBi_2Cl_{12}$ LDP NCs (the sample collected at 24 hr of the LDP transformation reaction) (b).

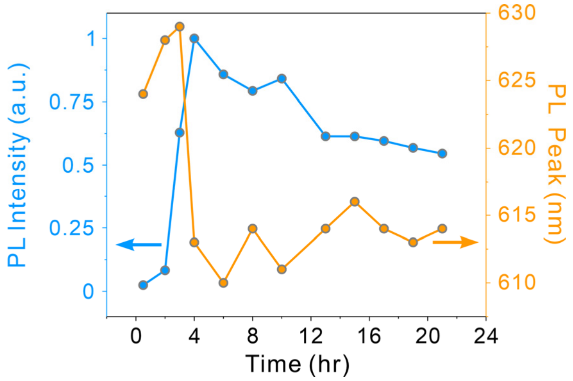

Figure S21. Summary of the evolutions of the PL peak wavelength and the relative PL peak intensity (normalized by the absorbance at excitation wavelength).

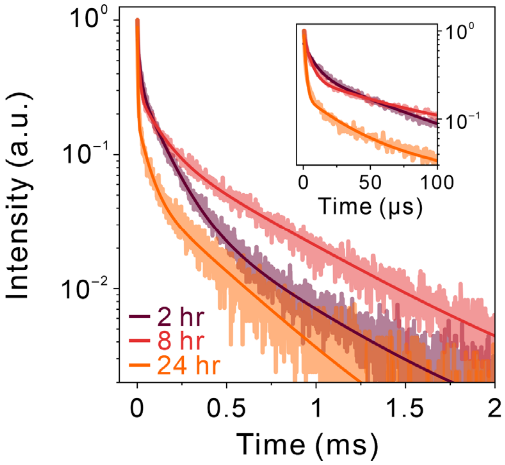

Figure S22. PL lifetime decays for the LDP transformation reaction. The experimental data are shown in translucent lines and the fitted data are shown in solid lines. Inset: zoomed-in time-resolved PL spectra.

Table S11. Tri-exponential fitting parameters of the time-resolved PL spectroscopy of the transformation reaction into the LDP NCs with different reaction time.

Reaction time (hr)	τ (μs)	Rel. percentage (%)	τ_{avg} (μs)
2	15.1 ± 0.4	9.5 ± 0.3	250 ± 4
	123 ± 2	55.1 ± 0.7	
	509 ± 7	35.4 ± 0.7	
8	9.1 ± 0.4	6.6 ± 0.3	435 ± 6
	111 ± 4	21.6 ± 0.8	
	572 ± 6	71.8 ± 0.8	
17	2.8 ± 0.1	9.9 ± 0.4	252 ± 4
	56 ± 2	24.8 ± 0.8	
	363 ± 4	65.3 ± 0.8	

References

1 H. Yang, T. Cai, E. Liu, K. Hills-Kimball, J. Gao and O. Chen, *Nano Res.*, 2020, **13**, 282-291.

2 S. E. Creutz, E. N. Crites, M. C. De Siena and D. R. Gamelin, *Nano Lett.*, 2018, **18**, 1118-1123.

3 H. Yang, W. Shi, T. Cai, K. Hills-Kimball, Z. Liu, L. Dube and O. Chen, *Nanoscale*, 2020, **12**, 23191-23199.

Author Contributions

H.Y. and O.C. conceived and designed the experiments. H.Y. performed NC synthesis and the transformation reaction. T.C. performed DP NC synthesis and XPS measurements. H.Y. and L.D. performed optical spectra measurements. H.Y. performed XRD, TEM, Raman, and EPR measurements. O.C. supervised the entire projects. The manuscript was written through the contributions of all authors. All authors have given approval to the final version.
